# Joint association between pan-immune inflammation value and physical pain with self-perception of patients in rheumatoid arthritis: a retrospective cohort study employing traditional statistics and interpretable machine learning

**DOI:** 10.3389/fimmu.2025.1711081

**Published:** 2025-12-18

**Authors:** Yang Li, Jian Liu, Yue Sun, Yuedi Hu, Jianting Wen, Xueni Cheng, Shengfeng Liu

**Affiliations:** 1 Department of Rheumatology, The First Affiliated Hospital of Anhui University of Chinese Medicine, Hefei, Anhui, China; 2 Anhui Provincial Key Laboratory of Applied Basic and Clinical Translational Research in Traditional Chinese Medicine Rheumatology, Hefei, Anhui, China; 3 First Clinical Medical School, Anhui University of Chinese Medicine, Hefei, Anhui, China

**Keywords:** cohort, pan-immune inflammation value, physical pain, rheumatoid arthritis, self-perception of patients

## Abstract

**Background:**

Patients with rheumatoid arthritis (RA) frequently experience increased physical pain and impaired self-perception. However, the combined impact of the pan-immune-inflammation value (PIV) and the visual analogue scale (VAS), representing subjective pain on SPP has not been thoroughly investigated.

**Methods:**

This retrospective cohort study included baseline clinical data of patients with RA admitted to the First Affiliated Hospital of Anhui University of Chinese Medicine. The study outcome was defined as SPP scores, while the exposure variables were the initial PIV and VAS values. The associations were evaluated using Spearman’s correlation, restricted cubic splines, and multivariate logistic regression. Interaction effects were evaluated by incorporating product terms, and potential mechanisms were explored through mediation analysis. Additionally, Extreme gradient boosting (XGBoost) models were developed for SPP outcomes, followed by the Shapley Additive exPlanations (SHAP) method to explain predicted values.

**Results:**

The analysis included 1,426 patients with RA. Patients with concurrent high levels of PIV and VAS exhibited significantly elevated inflammatory markers and the poorest SPP scores (all P < 0.001). Higher levels of PIV and VAS were independently associated with increased risks of deterioration across multiple SPP domains, with significant multiplicative and additive interactions observed. Compared with the low PIV and low VAS group, the high PIV and high VAS group demonstrated the greatest risk of decline in physical functioning, bodily pain, vitality, role-emotional, mental health, the Chinese Patient-Reported Activity Index for RA (CPRI-RA), Syndrome Score of Dampness-Heat, and Syndrome Score of Dampness Stagnancy due to Spleen Deficiency. Mediation analysis revealed VAS partially mediated the association between PIV and several SPP outcomes. The XGBoost models integrating PIV and VAS achieved superior predictive performance for social functioning and CPRI-RA (AUC = 0.755 and 0.748, respectively). SHAP analysis identified VAS and PIV as the most important predictive features.

**Conclusion:**

PIV and VAS are independent and synergistic risk factors for impaired SPP in RA patients. Combined assessment of PIV and VAS improves the prediction of SPP deterioration and may serve as a valuable strategy for optimizing clinical management.

## 1 Introduction

Rheumatoid arthritis (RA) is a systemic autoimmune disease characterized by chronic synovitis that can lead to progressive joint destruction, functional impairment, and systemic complications (1). The global prevalence of RA is approximately 0.5–1%, with women being nearly three times more likely to develop the disease than men, and its incidence continues to rise (2). It is estimated that by 2050, the number of individuals affected by RA worldwide may reach 31.7 million (3). RA not only causes joint deformities and physical disabilities but also substantially increases the risk of comorbidities, including cardiovascular events and interstitial lung disease (4). Consequently, it leads to a marked decline in quality of life, reduced work capacity, and psychological conditions such as depression and anxiety. Moreover, the complex etiology and highly active disease course of RA often result in poor prognosis and treatment challenges. Therefore, identifying modifiable risk factors and developing novel assessment methods are crucial for the early detection and targeted prevention of RA in high-risk populations.

For a long time, the disconnect between traditional inflammatory markers and patients’ subjective experiences has limited the scientific interpretation of disease activity in RA. The International Consortium for Health Outcomes Measurement (ICHOM) has proposed a set of patient-reported outcomes (PROs) for inflammatory arthritis, including RA, that focus on pain, activity limitations, fatigue, and the overall impact on emotional and physical health, and recommends their global implementation (5). The self-perception of patients (SPP) is derived from this standardized outcome assessment framework, integrating the Short Form 36 Health Survey (SF-36) (6), the Chinese Patient-Reported Activity Index for Rheumatoid Arthritis (CPRI-RA) (7), the Self-Rating Anxiety Scale (SAS) (8), and the Self-Rating Depression Scale (SDS) (9). To reflect the characteristics of traditional Chinese medicine (TCM) and dynamically track syndrome-related effects, an innovative TCM syndrome-specific assessment system was incorporated, encompassing three common syndrome dimensions in patients with RA: Syndrome Score of Dampness-Heat (SDH) (10), Syndrome Score of Dampness Stagnancy due to Spleen Deficiency (SDSSD) (11), and Syndrome Score of Blood Stasis (SBS) (12). This system represents a unique supplement and innovation to the clinical outcomes recommended by ICHOM. Previous studies have shown that patients with RA frequently exhibit abnormal SPP, manifested as anxiety, depression, and reduced quality of life, with the severity significantly correlated with inflammatory status and molecular alterations (13, 14). Notably, prior research has predominantly focused on objective disease activity or survival endpoints, largely overlooking the comprehensive perception and functional status of RA patients. Therefore, constructing a composite SPP outcome set may enable more sensitive detection of patients’ multidimensional symptom profiles, providing an important outcome indicator for assessing disease status in RA.

Inflammatory responses driven by multiple immune cells play a central role in initiating and amplifying pathological damage in RA (15). Traditional clinical markers such as erythrocyte sedimentation rate (ESR) and C-reactive protein (CRP) objectively reflect inflammation levels; however, they are easily affected by various factors, including infection and acute stress (16). The pan-immune inflammation value (PIV) is an emerging composite biomarker that quantifies the balance between pro-inflammatory (neutrophils, monocytes, platelets) and anti-inflammatory (lymphocytes) cell populations, providing a more comprehensive assessment of systemic immune-inflammatory status (17). In recent years, PIV has shown superior prognostic value in cancer and infectious diseases; however, studies investigating its association with clinical outcomes in RA remain limited (18, 19). Physical pain, the most prominent symptom of RA, is a principal driver of patients’ subjective distress, and the visual analogue scale (VAS) for pain assessment serves as the reference standard for pain evaluation (20). Although previous studies have established a link between inflammatory responses and chronic pain (21), how the comprehensive immune-inflammatory status represented by PIV and the subjective pain characterized by VAS interact synergistically remains unknown, representing a critical research gap. Currently, evidence integrating PIV and VAS into a unified predictive framework to elucidate the mechanisms underlying SPP deterioration in RA patients remains scarce.

To address this knowledge gap, this study innovatively proposes an “inflammation-pain dual-driver model,” which integrates objective inflammation represented by PIV and subjective pain measured by VAS, employing a combined analytical strategy of traditional statistics and explainable machine learning to predict the risk of multidimensional SPP deterioration. Traditional methods are powerful for establishing dose-response relationships and testing interactions but may overlook complex nonlinear patterns. In contrast, machine learning excels at capturing these intricate relationships and ranking feature importance, with further SHAP analysis ensuring clinical interpretability of the results. In summary, this study aims to transcend the conventional single-biomarker paradigm by comprehensively elucidating the biological mechanisms underlying the deterioration of multidimensional perceptions in RA patients—from association inference and mechanistic exploration to outcome prediction—thereby providing evidence-based support for clinical outcome assessment and individualized intervention in RA.

## 2 Materials and methods

### 2.1 Data sources and study population

This single-center, retrospective study was conducted in the Department of Rheumatology, First Affiliated Hospital of Anhui University of Chinese Medicine. The study was registered with the International Traditional Medicine Clinical Trial Registry (ITMCTR) on June 25, 2025 (registration number: ITMCTR2025001241). It was performed in full accordance with the ethical principles outlined in the Declaration of Helsinki, and all procedures complied with the relevant protocols and regulations specified therein. Ethical approval was obtained from the Ethics Committee of the First Affiliated Hospital of Anhui University of Chinese Medicine (Approval No. 2025AH-57).

**Figure 1:**
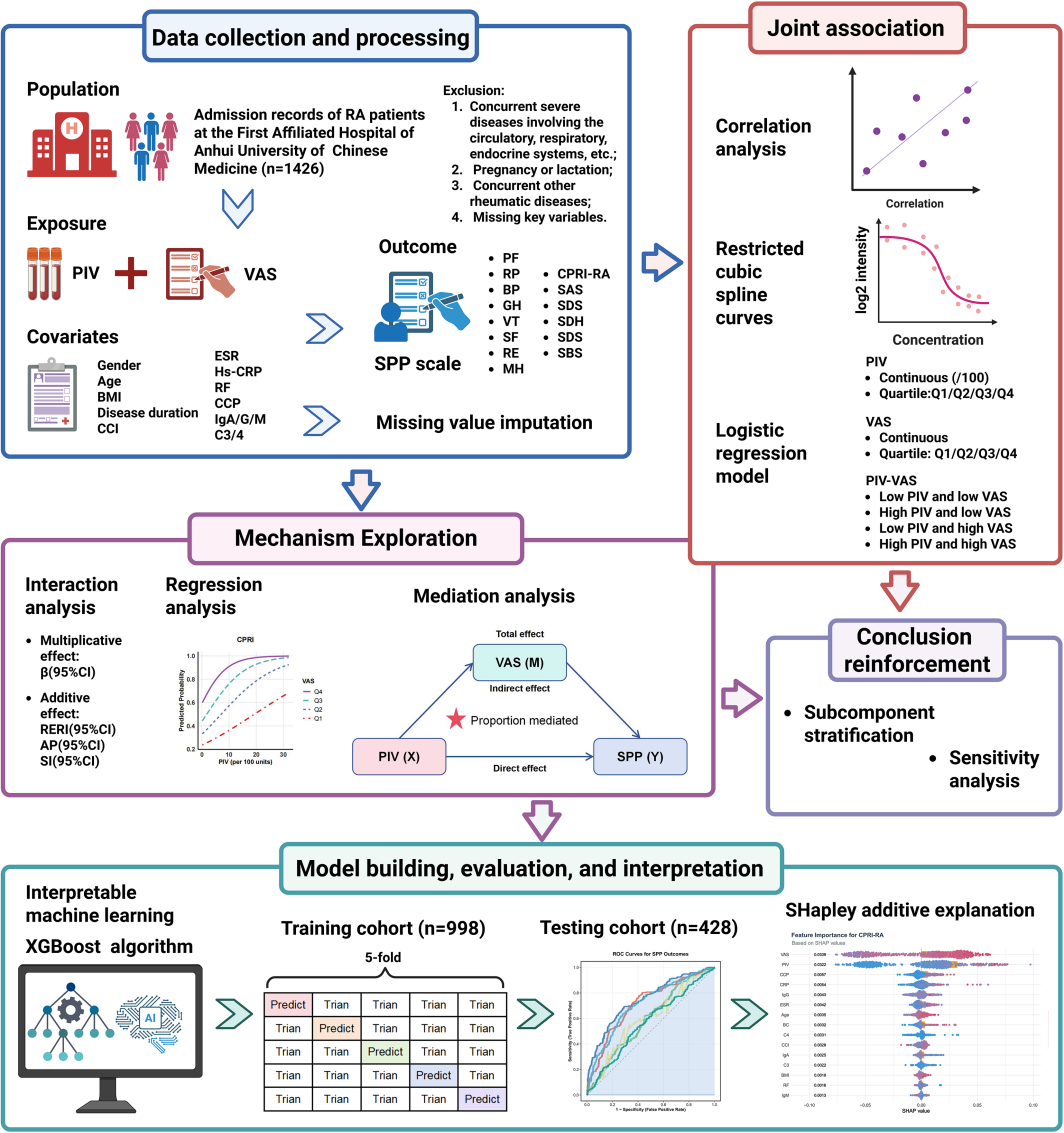
Research design and overall workflow of the study.

A retrospective review of clinical data was conducted for patients with RA admitted to the Department of Rheumatology, First Affiliated Hospital of Anhui University of Chinese Medicine, between August 2023 and June 2025. The diagnosis of RA was established based on the International Classification of Diseases, 10th Revision code M06.900. All patients fulfilled the 2010 classification criteria for RA proposed by the American College of Rheumatology and the European League Against Rheumatism (22). Participants were excluded according to the following criteria: (1) the presence of severe comorbidities affecting the cardiovascular, hematologic, respiratory, or endocrine systems; (2) pregnancy or breastfeeding; (3) presence of other rheumatic diseases such as systemic lupus erythematosus or ankylosing spondylitis; and (4) missing key variables required for analysis, including blood cell counts for PIV calculation, VAS, or SPP core questionnaire data. Ultimately, 1,426 participants were included in the final analysis. The detailed study procedures are illustrated in **Figure 1**.

### 2.2 Exposure measurement

The exposure variables in this study were the initial PIV and VAS scores. The PIV was calculated using the following formula: PIV = (neutrophil count × monocyte count × platelet count)/lymphocyte count. Blood cell data were obtained from the first laboratory test conducted after hospital admission, typically using fasting venous blood samples collected in the early morning. Pain intensity was evaluated using a 0–10 cm visual analogue scale, where 0 indicated no pain and 10 indicated the most severe pain. Assessments were conducted by trained healthcare professionals within 24 hours of admission in a quiet ward environment, and patients were asked to “mark the average pain level over the past 24 hours”.

### 2.3 Definition of study outcomes

The SPP scales, which have a multidimensional structure, were used as the outcome measures in this study. The SF-36 is widely recommended as an important reference tool for evaluating physical and mental health in clinical studies of RA (23, 24). It assesses eight dimensions: physical functioning (PF), role-physical (RP), bodily pain (BP), general health (GH), vitality (VT), social functioning (SF), role-emotional (RE), and mental health (MH). Each dimension of the SF-36 is scored on a scale from 0 to 100 as a general health rating index, with scores of 50 or higher considered within the normal range. The CPRI-RA is a population-specific RA–patient-reported outcome scale designed to quantitatively assess disease activity, providing an objective supplementary tool for evaluating efficacy in RA clinical trials (7, 25). The scale includes 11 items, each rated from 0 to 3 according to four severity levels (ranging from no symptoms to severe symptoms), with the total score calculated according to a predefined formula. The SDS and SAS are standardized tools for assessing the psychological status of patients with RA and are widely used internationally (26). Each scale uses a 4-point rating system, with total scores obtained by summing responses to 20 items based on symptom frequency. A total score of 50 or higher indicates the presence of depression or anxiety. In addition, the TCM syndrome scores—specifically, SDH, SDSSD, and SBS—are used to assess TCM-related syndromes. These correspond respectively to damp-heat syndrome (characterized by joint burning and swelling, red tongue with thick yellow coating), spleen deficiency with dampness (manifested by fatigue, loss of appetite, and loose stools), and blood stasis syndrome (associated with fixed joint pain) (12, 27). These scores convert patients’ self-reported clinical symptoms into standardized quantitative indicators through weighted scoring of primary and secondary symptom items.

Within 24 hours of admission, two rheumatologists or trained researchers conducted face-to-face interviews with patients. During questionnaire administration, the researchers provided detailed and easily understandable instructions to ensure accurate completion. Patients were instructed to select the options corresponding to their current condition for each question. After the questionnaire assessments were completed, two researchers independently entered the data, which were subsequently verified by a third-party auditor for logical consistency and completeness.

### 2.4 Assessment of covariates

In this study, the covariates considered included sociodemographic characteristics, disease-related factors, and laboratory indicators. Sociodemographic characteristics comprised sex and age. Participants underwent standardized anthropometric measurements to determine height and weight. Body mass index (BMI) was calculated using the formula: BMI (kg/m²) = weight (kg)/[height (m)]^2^. Disease duration and comorbidities were defined based on self-reported information obtained at admission. The Charlson–Deyo Comorbidity Index (CCI) applies a weighted scoring system that accounts for the presence and relative severity of 17 predefined comorbid conditions and was used in this study to quantify participants’ comorbidity burden (28). In addition, based on clinical relevance, the following laboratory indicators were included: ESR, high-sensitivity C-reactive protein (hs-CRP), rheumatoid factor (RF), anti-cyclic citrullinated peptide antibody (anti-CCP), immunoglobulin A (IgA), immunoglobulin G (IgG), immunoglobulin M (IgM), complement component 3 (C3), and complement component 4 (C4).

### 2.5 Statistical analysis

#### 2.5.1 Descriptive analysis

The Shapiro–Wilk test was used to assess the normality of all continuous variables. For the description of baseline characteristics, normally distributed continuous variables were presented as the mean ± standard deviation, whereas non-normally distributed variables were summarized as the median and interquartile range. Categorical variables were expressed as counts and percentages [n (%)]. Differences in baseline characteristics between groups were examined using analysis of variance, the Kruskal–Wallis rank-sum test for continuous variables, and the chi-square (χ^2^) test for categorical variables. Assuming that data were missing at random, multiple imputation was applied to handle missing data and minimize variability resulting from missingness. **Supplementary Table 1** provides detailed information on missing data (6.1%; 87 of 1,426 participants) and the imputation methods employed. Descriptive analysis and data preprocessing were primarily conducted utilizing SPSS software (version 26.0).

#### 2.5.2 Correlation analysis

Spearman’s rank correlation is a nonparametric method that assesses the strength and direction of the monotonic association between two continuous variables. The Mantel test, proposed by Nathan Mantel, evaluates the correlation between two distance matrices (29). In this study, Spearman’s rank correlation and the Mantel test were used to explore associations among all variables and between variables and SPP outcomes. Corresponding heatmaps, scatter plots, and network diagrams were generated to visualize these relationships. These results were obtained in R 4.3.2 using the “Hmisc” (v5.2-4) and “linkET” (v0.1.0) packages.

#### 2.5.3 Restricted cubic spline analysis

To explore potential dose–response relationships (linear or nonlinear) between systemic inflammatory indicators and SPP outcomes, restricted cubic spline (RCS) regression analyses were performed for the PIV, VAS, and each SPP indicator individually. The SPP scores, serving as dependent variables, were converted from continuous to binary variables according to their predefined thresholds or median values. RCS curves were generated using logistic regression models, with the number of knots determined based on the minimum Akaike information criterion (AIC) value. These models are primarily implemented using the “rms” (v8.1-0) package in R 4.3.2.

#### 2.5.4 Logistic regression models

To evaluate the associations between PIV, VAS, and variability in SPP outcomes—as well as their combined effects—multiple stepwise logistic regression models with incremental adjustments were constructed. Prior to model fitting, multicollinearity among covariates was assessed to ensure model stability. The SPP outcome indicators were dichotomized according to their predefined thresholds or median values. The independent variables, PIV and VAS, were analyzed both as continuous variables and as categorical variables divided into quartiles for further comparison. Because PIV exhibited a wide range and a right-skewed distribution, it was scaled by dividing by 100 to improve model convergence and enhance the interpretability of the regression coefficients. Participants were further categorized into four exposure groups based on the median values of PIV and VAS: low PIV/low VAS, high PIV/low VAS, low PIV/high VAS, and high PIV/high VAS. This grouping enabled evaluation of joint associations and potential interaction effects. Three hierarchical logistic regression models were established: Model 1 was unadjusted (crude model); Model 2 adjusted for age, sex, and BMI; and Model 3 additionally adjusted for disease-related variables based on Model 2, including disease duration, CCI, ESR, hs-CRP, RF, anti-CCP, IgA, IgG, IgM, C3, and C4. Results were expressed as odds ratios (ORs) with 95% confidence intervals (CIs) for all three models. Forest plots were used to visualize the results, with Model 3 serving as the primary presentation. Additionally, to account for multiple testing, all P-values for Model 3 underwent false discovery rate (FDR) correction using the Benjamini-Hochberg method.

#### 2.5.5 Interaction, moderation, and mediation analyses

To test the hypothesis regarding the interaction and intrinsic mechanistic association between PIV and VAS, we performed interaction, moderation, and mediation analyses. First, multiplicative interaction was assessed by incorporating the product term between PIV and VAS into a logistic regression model. Three indices were calculated to evaluate biological additive interaction (30): the relative excess risk due to interaction (RERI), the attributable proportion (AP) due to interaction, and the synergy index (SI). Subsequently, moderation analysis was conducted to examine the moderating effect of different VAS levels on the relationship between PIV and SPP outcomes. Finally, mediation analysis was performed to determine whether VAS mediated the association between PIV and SPP outcomes. All analyses were conducted after full adjustment for covariates, including age, gender, BMI, disease duration, CCI, ESR, hs-CRP, RF, CCP, IgA, IgG, IgM, C3, and C4). These analyses were primarily implemented in R 4.3.2 using the “mediation” (v4.5.1), “interactionR” (v0.1.7), and “interactions” (v1.2.0) packages.

#### 2.5.6 Subgroup analysis

Subgroup analyses were performed to evaluate potential heterogeneity in the combined effect of PIV and VAS on the risk of SPP deterioration. Participants were stratified according to baseline characteristics, including gender (male/female), age (<60/≥60 years), BMI (<24/≥24 kg/m^2^), disease duration (<9/≥9 years), and CCI (<4/≥4). These variables were used as effect modifiers in repeated logistic regression models to investigate whether demographic and disease-related factors moderated the association between PIV–VAS combinations and SPP outcomes. The statistical significance of interaction terms was further assessed using likelihood ratio tests. All results were obtained using R 4.3.2, involving the “tidyverse” (v2.0.0), “broom” (v1.0.10), “lmtest” (v0.9-40), and “forestplot” (v3.1.7) packages.

#### 2.5.7 Sensitivity analysis

To evaluate the robustness and validity of the study findings, several sensitivity analyses were performed: (1) The primary analyses were repeated using K-means clustering to categorize participants into different levels, thereby minimizing the potential influence of median-based grouping on the results; (2) All SPP outcomes were converted into ordinal four-category variables based on quartiles and analyzed using ordinal logistic regression, with three stepwise-adjusted models consistent with the main analysis; (3) Continuous covariates were incorporated into the model using a generalized additive model (GAM), implemented using the “mgcv” (v1.9-3) package in R 4.3.2, and the AIC and Bayesian information criterion (BIC) were calculated to compare model fit (31); (4) The E-value was calculated to assess the potential impact of unmeasured confounders on the association between combined PIV–VAS exposure and the risk of SPP variation (32).

#### 2.5.8 Machine learning modeling, evaluation, and interpretation

For the machine learning analysis predicting SPP outcomes, the entire cohort was randomly divided into a training cohort and a testing cohort at a 7:3 ratio. This split follows a common convention in predictive modeling, ensuring adequate data for model training while retaining a sufficiently large independent set for robust evaluation of model generalizability. The training cohort was used for model development, whereas the testing cohort served for model evaluation. First, the 16 feature variables, identified through clinical relevance and preliminary analyses, were fed into the Extreme Gradient Boosting (XGBoost) algorithm, with model training and hyperparameter tuning for each SPP outcome performed via a five-fold cross-validated grid search (33). Second, the discriminative ability, calibration, and clinical net benefit of all outcome models were evaluated in the testing cohort. Discriminative metrics included accuracy, sensitivity (SEN, recall), specificity (SPE), area under the receiver operating characteristic curve (AUC), positive likelihood ratio, negative likelihood ratio, positive predictive value (PPV, precision), negative predictive value, and F1 score. Model performance and clinical utility were visualized using receiver operating characteristic (ROC) curves, calibration plots, and decision curve analysis (DCA). Third, the net reclassification improvement and integrated discrimination improvement indices were used to assess the incremental predictive value of PIV, VAS, and their combined models compared with traditional models. Finally, to interpret the internal mechanisms of the complex models, SHAP values were calculated to quantify the contribution of each feature to outcome prediction (34). Dependence plots were generated to illustrate the marginal effects of individual features—particularly PIV and VAS—on the model outputs. All procedures were performed in R 4.3.2, utilizing the following package versions: “xgboost” (v1.7.11.1) for modeling, “pROC” (v1.19.0.1) for performance evaluation, and “SHAPforxgboost” (v0.1.3) along with “shapviz” (v0.10.3) for SHAP-based interpretation.

## 3 Results

### 3.1 Baseline characteristics of the study population

A total of 1,426 participants who met the inclusion criteria were enrolled in this study. Overall, participants exhibited a relatively widespread decline in SPP outcomes, as illustrated in **Supplementary Figure 1**. Participants were categorized into four subgroups based on the median values of PIV and VAS: low PIV and low VAS (n = 441), high PIV and low VAS (n = 311), low PIV and high VAS (n = 272), and high PIV and high VAS (n = 402). The baseline characteristics of the participants are summarized in **Table 1**. Significant differences were observed across groups in demographic and clinical characteristics, including sex, age, disease duration, and CCI. Compared with participants in the low PIV and low VAS group, those in the high PIV and high VAS group exhibited markedly higher levels of ESR, hs-CRP, RF, IgA, C3, and C4. In particular, participants with high PIV and high VAS exhibited poorer SPP outcome scores, characterized by substantially reduced scores for PF, RP, BP, GH, VT, SF, RE, and MH along with significantly elevated scores for CPRI-RA, SAS, SDS, SDH, and SDSSD. **Supplementary Tables 2, 3** further summarize the baseline characteristics of participants stratified by quartiles of PIV and VAS, respectively.

**Table 1:** Clinical characteristics of the study population.

Characteristic	Overall	Low PIV and low VAS	High PIV and low VAS	Low PIV and high VAS	High PIV and high VAS	P value
Participants	1426	441	311	272	402	
Gender, n (%)						<0.001
Male	251 (17.6%)	55 (12.5%)	66 (21.2%)	40 (14.7%)	90 (22.4%)	
Female	1,175 (82.4%)	386 (87.5%)	245 (78.8%)	232 (85.3%)	312 (77.6%)	
Age (years)	58.0 (51.0, 68.0)	57.0 (51.0, 67.0)	58.0 (51.0, 69.0)	58.0 (50.0, 67.8)	60.0 (53.0, 69.0)	0.007
Age (years), n (%)						0.005
<60	788 (55.3%)	265 (60.1%)	172 (55.3%)	157 (57.7%)	194 (48.3%)	
≥60	638 (44.7%)	176 (39.9%)	139 (44.7%)	115 (42.3%)	208 (51.7%)	
BMI (kg/m^2^)	22.21 (20.31, 23.94)	22.23 (20.44, 23.97)	22.14 (19.91, 24.22)	22.23 (20.31, 23.73)	22.23 (20.38, 23.88)	0.962
Disease duration (years)	9.2 (3.8, 16.0)	9.0 (3.0, 15.0)	8.0 (3.0, 15.0)	10.8 (5.9, 17.0)	9.0 (3.3, 15.5)	0.006
CCI (score)	4.0 (3.0, 6.0)	4.0 (3.0, 5.0)	4.0 (3.0, 5.0)	5.0 (4.0, 6.0)	5.0 (4.0, 6.0)	<0.001
ESR (mm/h)	36.00 (17.00, 56.00)	23.00 (11.00, 44.00)	40.00 (23.00, 62.00)	30.00 (17.00, 49.75)	45.05 (27.11, 69.25)	<0.001
Hs-CRP (mg/L)	12.26 (3.23, 35.19)	4.89 (1.66, 13.78)	23.64 (8.00, 52.25)	7.12 (2.04, 23.97)	26.44 (7.69, 54.04)	<0.001
RF (KIU/L)	104.70 (38.90, 259.30)	81.70 (32.80, 206.50)	121.80 (47.60, 308.50)	83.95 (34.00, 230.75)	118.05 (54.10, 301.00)	<0.001
CCP (U/ml)	88.50 (15.63, 247.87)	101.00 (14.05, 257.66)	75.50 (19.50, 247.00)	92.70 (16.13, 250.25)	87.40 (12.77, 222.75)	0.915
IgA (g/L)	2.81 (2.06, 3.74)	2.66 (1.96, 3.64)	2.94 (2.06, 3.97)	2.73 (2.07, 3.59)	2.89 (2.20, 3.86)	0.028
IgG (g/L)	11.58 (9.42, 14.42)	11.73 (9.73, 15.00)	11.70 (9.65, 14.25)	11.78 (9.24, 14.42)	11.13 (9.02, 14.05)	0.020
IgM (g/L)	1.25 (0.91, 1.72)	1.23 (0.91, 1.74)	1.25 (0.97, 1.73)	1.30 (0.87, 1.88)	1.27 (0.89, 1.67)	0.639
C3 (g/L)	1.22 (1.08, 1.37)	1.17 (1.03, 1.32)	1.29 (1.16, 1.45)	1.16 (1.03, 1.28)	1.27 (1.13, 1.42)	<0.001
C4 (g/L)	0.30 (0.24, 0.37)	0.29 (0.22, 0.35)	0.32 (0.25, 0.39)	0.28 (0.23, 0.35)	0.31 (0.24, 0.39)	<0.001
PIV	288.95 (161.19, 522.14)	146.48 (97.85, 211.92)	484.47 (360.75, 739.59)	189.08 (121.97, 236.96)	545.94 (404.63, 832.68)	<0.001
VAS (cm)	6.20 (5.50, 7.00)	5.50 (5.00, 6.00)	5.60 (5.20, 6.00)	7.00 (6.83, 8.00)	7.10 (7.00, 8.00)	<0.001
PF (score)	30.00 (25.00, 40.00)	40.00 (30.00, 55.00)	35.00 (25.00, 40.00)	30.00 (20.00, 35.00)	25.00 (20.00, 30.00)	<0.001
RP (score)	0.00 (0.00, 25.00)	25.00 (0.00, 25.00)	0.00 (0.00, 25.00)	0.00 (0.00, 25.00)	0.00 (0.00, 25.00)	<0.001
BP (score)	31.00 (22.00, 41.00)	41.00 (31.00, 41.00)	32.00 (30.99, 41.00)	31.00 (22.00, 41.00)	22.00 (22.00, 32.00)	<0.001
GH (score)	30.00 (20.00, 35.00)	30.00 (25.00, 40.00)	30.00 (25.00, 35.00)	25.00 (15.00, 35.00)	25.00 (15.00, 35.00)	<0.001
VT (score)	40.00 (30.00, 50.00)	45.00 (35.00, 50.00)	45.00 (35.00, 50.00)	35.00 (25.00, 45.00)	35.00 (20.00, 40.00)	<0.001
SF (score)	50.00 (37.50, 62.50)	50.00 (50.00, 62.50)	50.00 (50.00, 62.50)	37.50 (25.00, 50.00)	37.50 (25.00, 50.00)	<0.001
RE (score)	33.33 (0.00, 33.33)	33.33 (0.00, 66.66)	33.33 (0.00, 33.33)	0.00 (0.00, 33.33)	0.00 (0.00, 33.33)	<0.001
MH (score)	44.00 (36.00, 52.00)	48.00 (36.00, 56.00)	48.00 (40.00, 56.00)	40.00 (28.00, 48.00)	36.00 (24.00, 48.00)	<0.001
CPRI-RA (score)	9.86 (8.43, 11.18)	8.78 (7.59, 9.96)	9.32 (8.25, 10.39)	10.05 (8.78, 11.38)	11.15 (9.96, 12.31)	<0.001
SAS (score)	53.75 (48.75, 58.75)	52.50 (48.75, 56.25)	52.50 (50.00, 56.25)	53.75 (47.50, 61.25)	55.00 (48.45, 61.25)	<0.001
SDS (score)	61.25 (56.25, 66.25)	58.75 (55.00, 62.50)	60.00 (56.25, 62.50)	63.75 (57.50, 71.25)	65.00 (59.69, 73.75)	<0.001
SDH (score)	15.0 (12.0, 19.0)	14.0 (11.0, 17.0)	15.0 (12.0, 17.0)	17.0 (12.0, 20)	17.0 (15.0, 21.0)	<0.001
SDSSD (score)	14.0 (10.0, 18.0)	11.0 (8.0, 15.0)	13.0 (10.0, 16.0)	14.0 (10.0, 19.0)	17.0 (12.0, 20.0)	<0.001
SBS (score)	6.0 (4.0, 8.0)	5.0 (4.0, 7.0)	6.0 (4.0, 7.0)	6.0 (4.0, 8.0)	6.0 (4.0, 8.0)	0.122

Categorical variables were shown as n (percent, %). Continuous variables were shown as median (Interquartile range, IQR). P values for differences between groups were derived using a Pearson’s Chi-squared test or Kruskal-Wallis rank sum test.

### 3.2 Association between PIV, VAS, and SPP scores

**Figure 2:**
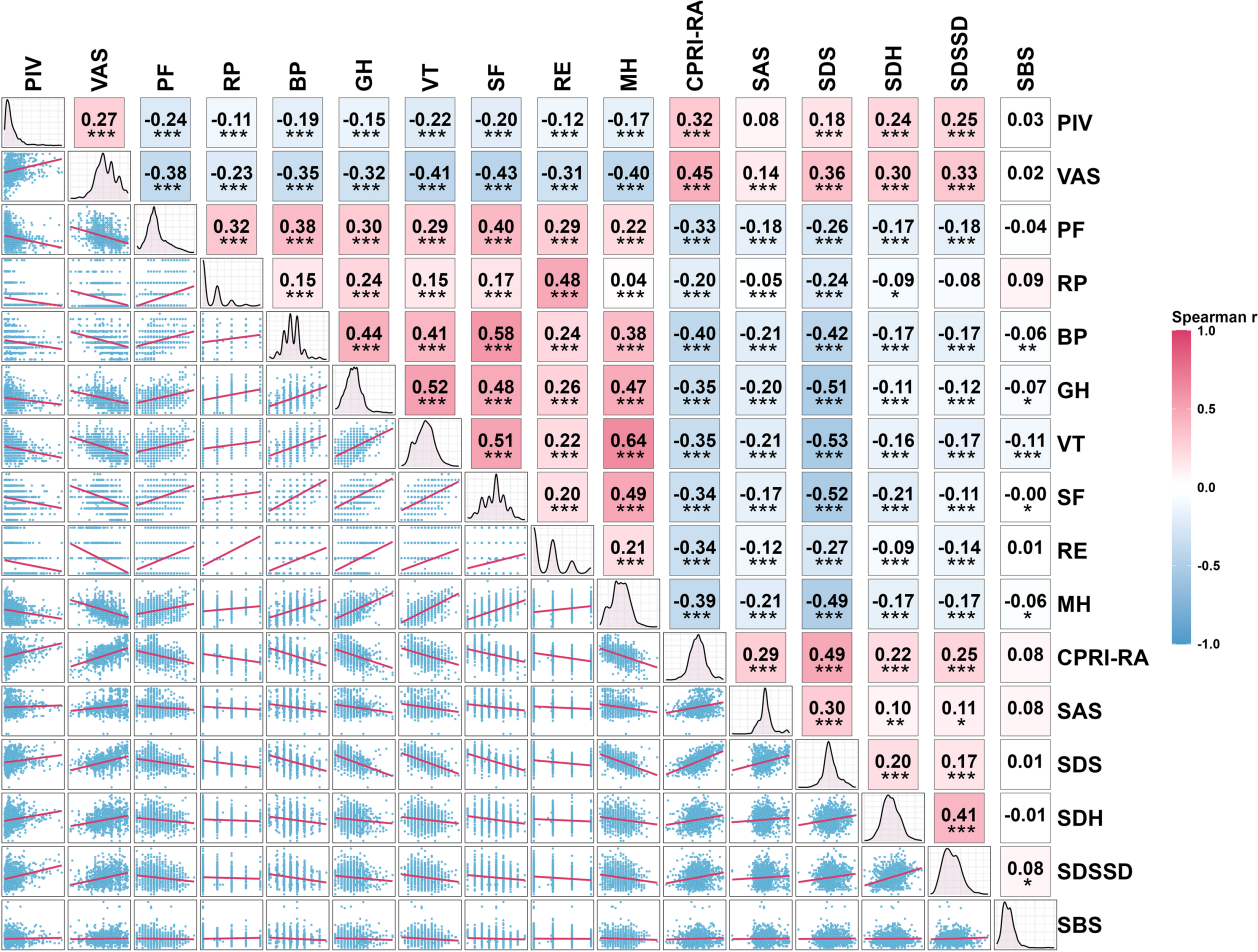
Correlation analysis between PIV, VAS, and SPP outcomes.

**Figure 3:**
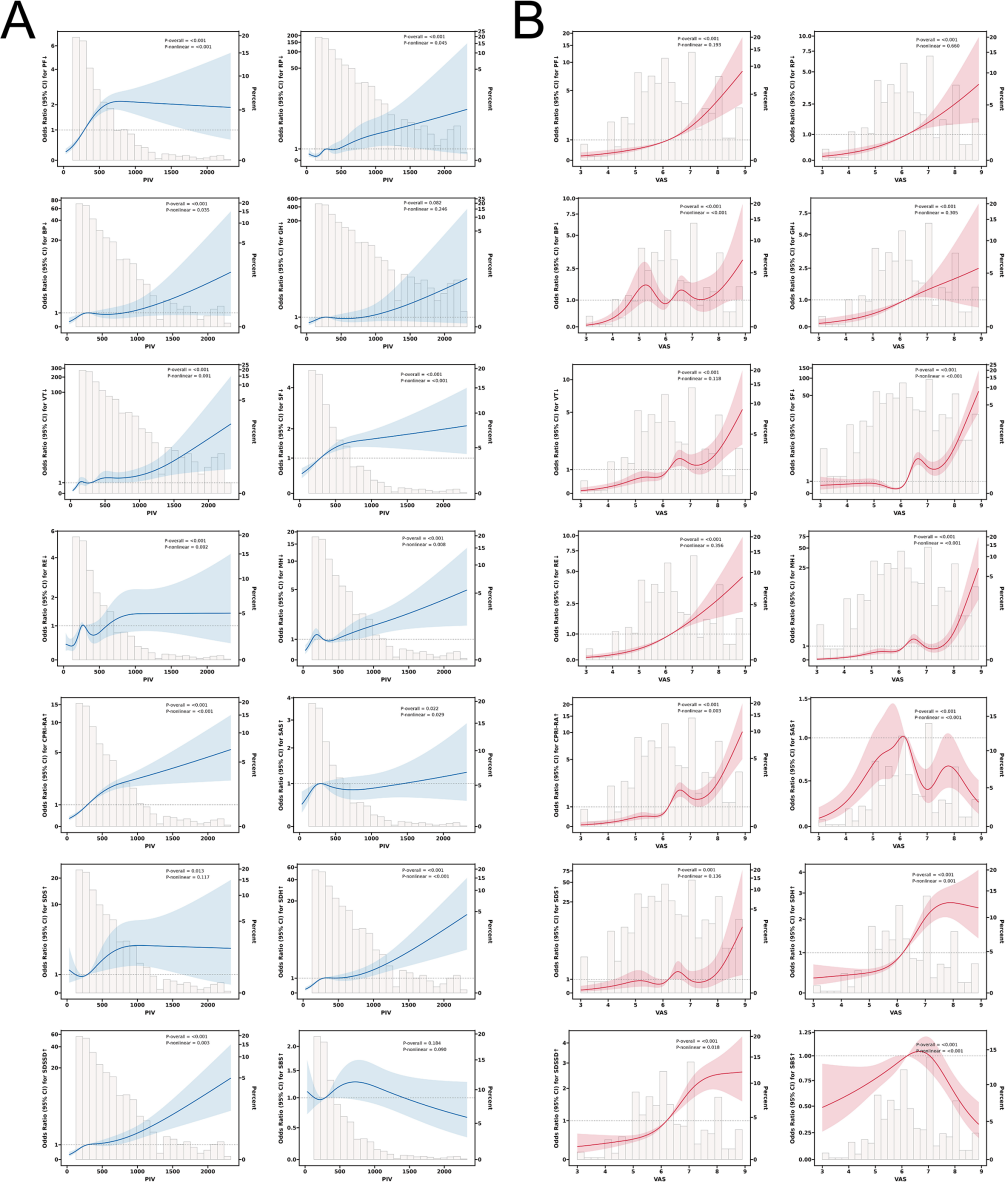
RCS analysis depicting the nonlinear relationships between **(A)** PIV and SPP outcomes and **(B)** VAS and SPP outcomes.

We further explored the associations between the PIV, VAS, and SPP outcomes in the study population. Spearman’s rank correlation analysis revealed that both PIV and VAS were negatively correlated with PF, RP, BP, GH, VT, SF, RE, and MH scores, while showing significant positive correlations with the CPRI-RA, SDS, SDH, and SDSSD scores (**Figure 2**). Additionally, PIV, VAS, and SAS scores were positively correlated with each other. RCS analysis was then applied to examine potential nonlinear associations between PIV or VAS and SPP outcomes. As shown in **Figure 3**, PIV demonstrated significant nonlinear relationships with PF, RP, BP, VT, SF, RE, MH, CPRI-RA, SAS, SDH, and SDSSD scores (P-overall < 0.05; P-nonlinear < 0.05) and a linear relationship with SDS scores (P-overall < 0.05; P-nonlinear > 0.05). For VAS, nonlinear correlations were observed with BP, SF, MH, CPRI-RA, SAS, SDH, SDSSD, and SBS scores (P-overall < 0.001; P-nonlinear < 0.05), whereas linear correlations were observed with PF, RP, GH, VT, RE, and SDS scores (P-overall < 0.01; P-nonlinear > 0.05). Notably, RCS curves for multiple SPP outcomes demonstrated partially similar visual patterns, indicating that elevated PIV or VAS levels increase the risk of deterioration in a broad, dose-response manner.

### 3.3 Independent effects of PIV or VAS on SPP deterioration outcomes

To ensure model stability, we first examined correlations among study variables to identify potential multicollinearity. Spearman’s rank correlation analysis and Mantel tests indicated no strong correlations among variables (**Supplementary Tables 4**, **5**; **Supplementary Figure 2**). The variance inflation factor values for all covariates were well below the critical threshold of 5, confirming the absence of multicollinearity (**Supplementary Figure 3**). Subsequently, multiple stepwise logistic regression analyses were performed to evaluate the independent associations between the PIV, VAS, and the risk of SPP deterioration (**Tables 2**, **3**). After full adjustment for potential confounders (Model 3), each 100-unit increase in PIV was associated with a 16%, 25%, 10%, 15%, 4%, 9%, 16%, 14%, and 16% higher risk of worsening in PF, RP, BP, VT, SF, RE, CPRI-RA, SDH, and SDSSD, respectively. Similarly, each one-point increase in VAS corresponded to an 88%, 80%, 97%, 75%, 103%, 90%, 102%, 55%, and 55% higher risk of deterioration in PF, RP, GH, VT, RE, MH, CPRI-RA, SDH, and SDSSD, respectively. When PIV and VAS were modeled as ordinal variables divided into quartiles, fully adjusted results showed that compared with the lowest quartile (Q1), participants in the highest quartile (Q4) exhibited 3.28-, 6.33-, 1.58-, 1.80-, 1.08-, 2.00-, 3.26-, 1.85-, and 2.19-fold higher odds of deterioration in PF, RP, BP, VT, SF, RE, CPRI-RA, SDH, and SDSSD, respectively. Notably, after multiple trend tests correction, all of the above associations remained statistically significant (all FDR q < 0.05). Overall, these findings indicate that PIV and VAS independently contributed to the risk of adverse SPP outcomes, each demonstrating significant and stable exposure–response relationships independent of the covariates included in the final adjusted model.

**Table 2:** Independent effects of PIV and SPP on deterioration outcomes.

Outcome	Exposure	Event/N	Model 1		Model 2		Model 3	
			OR(95%CI)	P value	OR(95%CI)	P value	OR(95%CI)	P value
PF	PIV, per 100 units	1151/1426	1.20(1.13, 1.27)	<0.001	1.19(1.12, 1.26)	<0.001	1.16(1.09, 1.24)	<0.001*
	PIV quartile, P for trend			<0.001		<0.001		<0.001*
	Q1	233/356	Ref		Ref		Ref	
	Q2	283/357	2.02(1.44, 2.83)	<0.001	2.00(1.42, 2.80)	<0.001	1.99(1.41, 2.83)	<0.001*
	Q3	313/357	3.76(2.56, 5.51)	<0.001	3.64(2.47, 5.35)	<0.001	3.57(2.37, 5.37)	<0.001*
	Q4	322/356	5.00(3.30, 7.58)	<0.001	4.70(3.09, 7.15)	<0.001	4.28(2.68, 6.83)	<0.001*
RP	PIV, per 100 units	1263/1426	1.25(1.15, 1.36)	<0.001	1.25(1.15, 1.36)	<0.001	1.25(1.14, 1.36)	<0.001*
	PIV quartile, P for trend			<0.001				<0.001*
	Q1	281/356	Ref		Ref		Ref	
	Q2	313/357	1.90(1.27, 2.85)	<0.001	1.91(1.27, 2.87)	0.002	1.98(1.31, 3.01)	0.001*
	Q3	326/357	2.81(1.79, 4.39)	<0.001	2.78(1.77, 4.35)	<0.001	2.92(1.82, 4.71)	<0.001*
	Q4	343/356	7.04(3.83, 13.00)	<0.001	6.98(3.77, 12.90)	<0.001	7.33(3.76, 14.31)	<0.001*
BP	PIV, per 100 units	1276/1426	1.10(1.04, 1.17)	<0.001	1.09(1.03, 1.16)	0.004	1.10(1.03, 1.18)	0.004*
	PIV quartile, P for trend			<0.001		0.001		0.001*
	Q1	298/356	Ref		Ref		Ref	
	Q2	326/357	2.05(1.29, 3.25)	<0.001	2.01(1.26, 3.20)	0.003	2.17(1.35, 3.49)	0.003*
	Q3	322/357	1.79(1.14, 2.80)	0.010	1.74(1.11, 2.74)	0.016	1.91(1.18, 3.09)	0.008*
	Q4	330/356	2.47(1.52, 4.03)	<0.001	2.36(1.44, 3.86)	<0.001	2.58(1.48, 4.50)	0.001*
VT	PIV, per 100 units	1020/1426	1.14(1.09, 1.19)	<0.001	1.14(1.09, 1.19)	<0.001	1.15(1.10, 1.21)	<0.001*
	PIV quartile, P for trend			<0.001		<0.001		<0.001*
	Q1	221/356	Ref		Ref		Ref	
	Q2	248/357	1.39(1.02, 1.90)	0.040	1.41(1.03, 1.93)	0.033	1.45(1.05, 2.01)	0.023*
	Q3	262/357	1.66(1.21, 2.28)	<0.001	1.66(1.20, 2.28)	0.002	1.72(1.23, 2.42)	0.002*
	Q4	290/356	2.68(1.91, 3.78)	<0.001	2.68(1.89, 3.80)	<0.001	2.80(1.89, 4.14)	<0.001*
SF	PIV, per 100 units	559/1426	1.07(1.04, 1.10)	<0.001	1.07(1.04, 1.10)	<0.001	1.04(1.01, 1.08)	0.005*
	PIV quartile, P for trend			<0.001		<0.001		0.001*
	Q1	100/356	Ref		Ref		Ref	
	Q2	134/357	1.54(1.12, 2.11)	0.010	1.53(1.11, 2.10)	0.009	1.41(1.02, 1.95)	0.037*
	Q3	142/357	1.69(1.24, 2.31)	<0.001	1.66(1.21, 2.28)	0.002	1.48(1.06, 2.06)	0.002*
	Q4	183/356	2.71(1.99, 3.70)	<0.001	2.62(1.91, 3.59)	<0.001	2.08(1.46, 2.95)	<0.001*
RE	PIV, per 100 units	1164/1426	1.10(1.05, 1.15)	<0.001	1.10(1.05, 1.15)	<0.001	1.09(1.04, 1.15)	0.001*
	PIV quartile, P for trend			<0.001		<0.001		<0.001*
	Q1	254/356	Ref		Ref		Ref	
	Q2	298/357	2.03(1.41, 2.91)	<0.001	2.06(1.43, 2.96)	<0.001	2.07(1.43, 2.99)	<0.001*
	Q3	298/357	2.03(1.41, 2.91)	<0.001	2.04(1.42, 2.94)	<0.001	2.04(1.39, 2.99)	<0.001*
	Q4	314/356	3.00(2.02, 4.46)	<0.001	3.05(2.04, 4.56)	<0.001	3.00(1.92, 4.70)	<0.001*
CPRI-RA	PIV, per 100 units	713/1426	1.17(1.13, 1.22)	<0.001	1.17(1.13, 1.22)	<0.001	1.16(1.131 1.20)	<0.001*
	PIV quartile, P for trend			<0.001		<0.001		<0.001*
	Q1	107/356	Ref		Ref		Ref	
	Q2	158/357	1.85(1.36, 2.51)	<0.001	1.85(1.36, 2.52)	<0.001	1.79(1.30, 2.45)	<0.001*
	Q3	207/357	3.21(2.36, 4.37)	<0.001	3.18(2.33, 4.34)	<0.001	2.99(2.16, 4.14)	<0.001*
	Q4	241/356	4.88(3.55, 6.70)	<0.001	4.81(3.49, 6.64)	<0.001	4.26(2.99, 6.08)	<0.001*
SDH	PIV, per 100 units	808/1426	1.15(1.11, 1.20)	<0.001	1.16(1.12, 1.20)	<0.001	1.14(1.10, 1.19)	<0.001*
	PIV quartile, P for trend			<0.001		<0.001		<0.001*
	Q1	147/356	Ref		Ref		Ref	
	Q2	195/357	1.71(1.27, 2.30)	<0.001	1.73(1.29, 2.33)	<0.001	1.57(1.15, 2.13	0.004*
	Q3	215/357	2.15(1.60, 2.90)	<0.001	2.18(1.62, 2.95)	<0.001	2.03(1.47, 2.79)	<0.001*
	Q4	251/356	3.40(2.49, 4.64)	<0.001	3.49(2.55, 4.78)	<0.001	2.85(2.00, 4.06)	<0.001*
SDSSD	PIV, per 100 units	719/1426	1.16(1.12, 1.20)	<0.001	1.15(1.11, 1.20)	<0.001	1.16(1.11, 1.21)	<0.001*
	PIV quartile, P for trend			<0.001		<0.001		<0.001*
	Q1	120/356	Ref		Ref		Ref	
	Q2	168/357	1.56(1.16, 2.11)	<0.001	1.56(1.16, 2.10)	0.004	1.52(1.11, 2.08)	0.009*
	Q3	189/357	1.98(1.47, 2.67)	<0.001	1.95(1.45, 2.64)	<0.001	1.99(1.44, 2.76)	<0.001*
	Q4	233/356	3.33(2.45, 4.53)	<0.001	3.26(2.39, 4.45)	<0.001	3.19(2.23, 4.56)	<0.001*

Model 1, unadjusted.

Model 2, adjusted for gender, age, and BMI.

Model 3, adjusted for variables in Model 2 plus disease duration, CCI, ESR, Hs-CRP, RF, CCP, IgA, IgG, IgM, C3, and C4.

*Significant after Benjamini-Hochberg correction for false discovery rate (FDR < 0.05).

**Table 3:** Independent effects of VAS and SPP on deterioration outcomes.

Outcome	Exposure	Event/N	Model 1		Model 2		Model 3	
			OR(95%CI)	P value	OR(95%CI)	P value	OR(95%CI)	P value
PF	VAS, per unit	1151/1426	1.81(1.61, 2.05)	<0.001	1.81(1.60, 2.04)	<0.001	1.88(1.65 2.14)	<0.001*
	VAS quartile, P for trend			<0.001		<0.001		<0.001*
	Q1	256/380	Ref		Ref		Ref	
	Q2	288/372	1.66(1.20, 2.30)	0.002	1.67(1.20, 2.31)	0.002	1.74(1.24, 2.44)	0.001*
	Q3	301/349	3.04(2.09, 4.41)	<0.001	3.03(2.09, 4.41)	<0.001	3.38(2.28, 5.00)	<0.001*
	Q4	306/325	7.80(4.68, 13.00)	<0.001	7.56(4.53, 12.62)	<0.001	8.36(4.91, 14.24)	<0.001*
RP	VAS, per unit	1263/1426	1.77(1.54, 2.05)	<0.001	1.77(1.53, 2.04)	<0.001	1.80(1.55, 2.08)	<0.001*
	VAS quartile, P for trend			<0.001		<0.001		<0.001*
	Q1	303/380	Ref		Ref		Ref	
	Q2	328/372	1.89(1.27, 2.83)	0.002	1.91(1.28, 2.86)	0.002	1.92(1.28, 2.90)	0.002*
	Q3	319/349	2.70(1.72, 4.24)	<0.001	2.68(1.71, 4.21)	<0.001	2.80(1.76, 4.45)	<0.001*
	Q4	313/325	6.63(3.54, 12.43)	<0.001	6.61(3.52, 12.42)	<0.001	6.86(3.59, 13.10)	<0.001*
GH	VAS, per unit	1353/1426	1.77(1.46, 2.15)	<0.001	1.78(1.46, 2.16)	<0.001	1.97(1.61, 2.42)	<0.001*
	VAS quartile, P for trend			<0.001		<0.001		<0.001*
	Q1	329/380	Ref		Ref		Ref	
	Q2	339/372	1.75(1.00, 3.06)	0.050	1.73(0.99, 3.03)	0.054	2.00(1.12, 3.56)	0.019*
	Q3	342/349	4.46(2.04, 9.74)	<0.001	4.49(2.06, 9.80)	<0.001	5.83(2.59, 13.13)	<0.001*
	Q4	343/325	2.47(1.52, 4.03)	<0.001	4.12(1.88, 9.01)	<0.001	6.14(2.67, 14.11)	<0.001*
VT	VAS, per unit	1020/1426	1.64(1.48, 1.82)	<0.001	1.65(1.48, 1.83)	<0.001	1.75(1.56, 1.96)	<0.001*
	VAS quartile, P for trend			<0.001		<0.001		<0.001*
	Q1	215/380	Ref		Ref		Ref	
	Q2	253/372	1.63(1.21, 2.20)	0.001	1.62(1.20, 2.19)	0.002	1.73(1.27, 2.35)	0.001*
	Q3	271/349	2.67(1.93, 3.68)	<0.001	2.70(1.95, 3.74)	<0.001	3.11(2.21, 4.37)	<0.001*
	Q4	281/325	4.90(3.36, 7.15)	<0.001	4.83(3.31, 7.06)	<0.001	5.69(3.81, 8.50)	<0.001*
RE	VAS, per unit	1164/1426	1.97(1.74, 2.24)	<0.001	1.97(1.74, 2.25)	<0.001	2.03(1.78, 2.33)	<0.001*
	VAS quartile, P for trend			<0.001		<0.001		<0.001*
	Q1	245/380	Ref		Ref		Ref	
	Q2	307/372	2.60(1.85, 3.66)	<0.001	2.61(1.86, 3.67)	<0.001	2.72(1.92, 3.85)	<0.001*
	Q3	309/349	4.26(2.88, 6.29)	<0.001	4.25(2.88, 6.29)	<0.001	4.51(3.01, 6.76)	<0.001*
	Q4	303/325	7.60(4.69, 12.28)	<0.001	7.62(4.71, 12.35)	<0.001	8.41(5.09, 13.90)	<0.001*
MH	VAS, per unit	959/1426	1.77(1.59, 1.96)	<0.001	1.76(1.59, 1.96)	<0.001	1.90(1.69, 2.12)	<0.001*
	VAS quartile, P for trend			<0.001		<0.001		<0.001*
	Q1	192/380	Ref		Ref		Ref	
	Q2	234/372	1.66(1.24, 2.22)	0.001	1.65(1.23, 2.21)	0.001	1.77(1.31, 2.39)	<0.001*
	Q3	251/349	2.51(1.84, 3.41)	<0.001	2.51(1.85, 3.42)	<0.001	2.91(2.11, 4.03)	<0.001*
	Q4	282/325	6.42(4.40, 9.38)	<0.001	6.31(4.32, 9.23)	<0.001	7.60(5.09, 11.35)	<0.001*
CPRI-RA	VAS, per unit	713/1426	1.99(1.80, 2.21)	<0.001	1.99(1.79, 2.21)	<0.001	2.02(1.81, 2.26)	<0.001*
	VAS quartile, P for trend			<0.001		<0.001		<0.001*
	Q1	106/380	Ref		Ref		Ref	
	Q2	142/372	1.60(1.17, 2.17)	0.003	1.60(1.18, 2.18)	0.003	1.63(1.18, 2.23)	0.002*
	Q3	218/349	4.30(3.15, 5.88)	<0.001	4.30(3.14, 5.87)	<0.001	4.43(3.20, 6.14)	<0.001*
	Q4	247/325	8.19(5.83, 11.49)	<0.001	8.14(5.79, 11.43)	<0.001	8.19(5.72, 11.72)	<0.001*
SDH	VAS, per unit	808/1426	1.69(1.53, 1.86)	<0.001	1.69(1.53, 1.86)	<0.001	1.55(1.40, 1.72)	<0.001*
	VAS quartile, P for trend			<0.001		<0.001		<0.001*
	Q1	145/380	Ref		Ref		Ref	
	Q2	184/372	1.59(1.19, 2.12)	0.002	1.59(1.19, 2.12)	0.002	1.44(1.07, 1.94)	0.017*
	Q3	230/349	3.13(2.31, 4.24)	<0.001	3.13(2.31, 4.24)	<0.001	2.68(1.96, 3.67)	<0.001*
	Q4	249/325	5.31(3.82, 7.39)	<0.001	5.34(3.83, 7.43)	<0.001	4.12(2.92, 5.81)	<0.001*
SDSSD	VAS, per unit	719/1426	1.66(1.51, 1.83)	<0.001	1.66(1.51, 1.82)	<0.001	1.55(1.40, 1.71)	<0.001*
	VAS quartile, P for trend			<0.001		<0.001		<0.001*
	Q1	119/380	Ref		Ref		Ref	
	Q2	166/372	1.77(1.31, 2.38)	<0.001	1.77(1.31, 2.38)	<0.001	1.66(1.22, 2.25)	0.001*
	Q3	202/349	3.01(2.23, 4.08)	<0.001	3.01(2.22, 4.08)	<0.001	2.66(1.94, 3.65)	<0.001*
	Q4	232/325	5.47(3.96, 7.56)	<0.001	5.38(3.89, 7.45)	<0.001	4.40(3.14, 6.17)	<0.001*

Model 1, unadjusted.

Model 2, adjusted for gender, age, and BMI.

Model 3, adjusted for variables in Model 2 plus disease duration, CCI, ESR, Hs-CRP, RF, CCP, IgA, IgG, IgM, C3, and C4.

*Significant after Benjamini-Hochberg correction for false discovery rate (FDR < 0.05).

### 3.4 Combined effects of PIV and VAS on SPP deterioration outcomes

**Figure 4:**
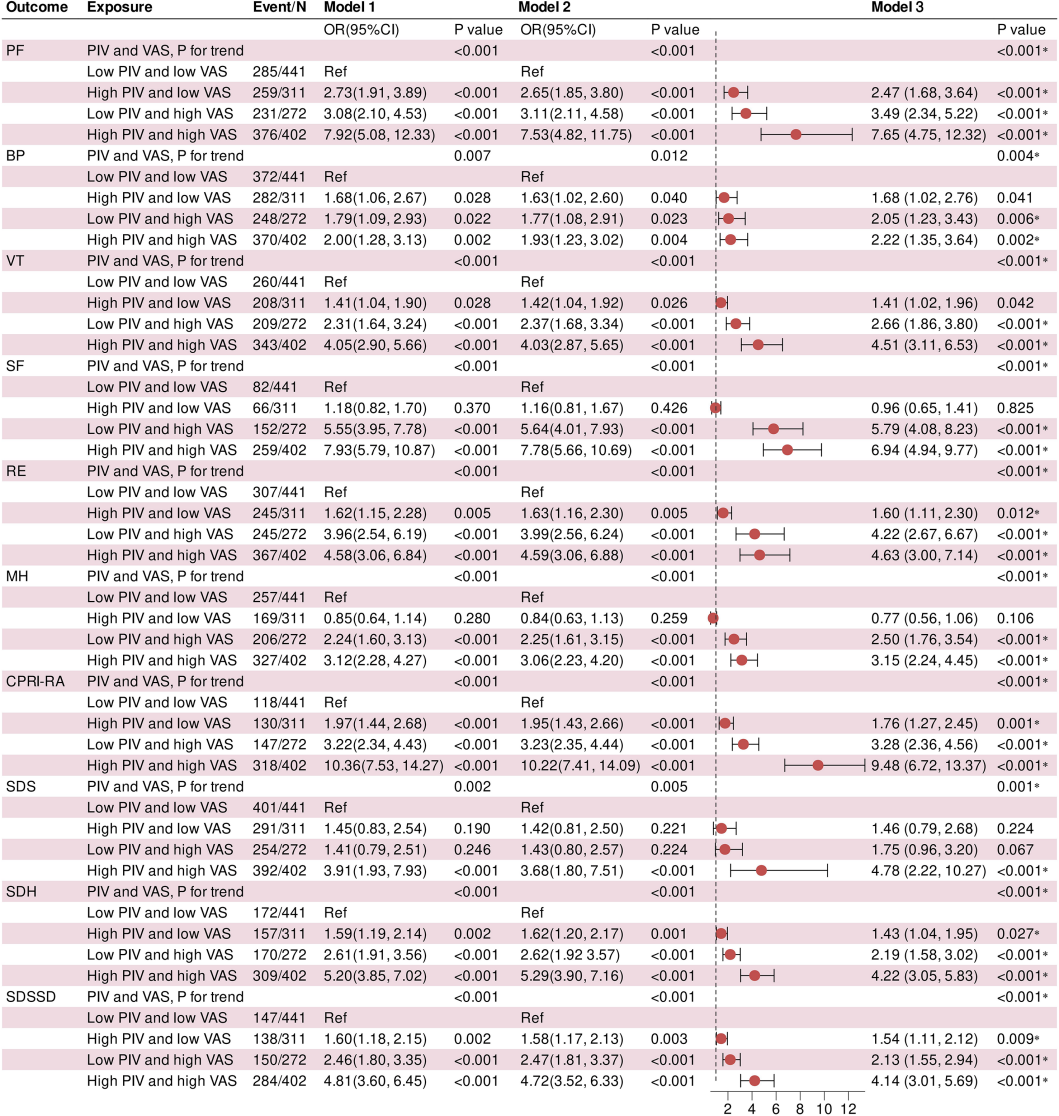
Risk effects of PIV combined with VAS on SPP deterioration outcomes. * Significant after Benjamini-Hochberg correction for false discovery rate (FDR < 0.05).

We further employed multiple stepwise-adjusted logistic regression models to quantify the combined effects of different PIV and VAS groupings on the risk of SPP deterioration (**Figure 4**). The four exposure groups were modeled as an ordered categorical variable, with the low PIV and low VAS group serving as the reference. After full adjustment (Model 3), individuals with high PIV alone, high VAS alone, and concurrent high PIV and high VAS exhibited significantly increased risks of deterioration in PF, BP, VT, RE, MH, CPRI-RA, SDH and SDSSD (all P < 0.05). For SF and SDS outcomes, although some associations did not reach statistical significance, similar trends were observed. Notably, participants in the high PIV/high VAS group consistently exhibited the greatest risk elevations across the above outcomes, with ORs of 7.65, 2.22, 4.51, 6.94, 4.63, 3.15, 9.48, 4.78, 4.22, and 4.14, respectively, compared with the reference group (all P < 0.001). The vast majority of associated trends remained statistically significant after multiple testing FDR correction (all FDR q < 0.05). These findings indicate a potential synergistic additive effect between elevated PIV and VAS levels on adverse SPP outcomes.

### 3.5 Interaction, moderation, and mediation effects of PIV and VAS on SPP deterioration outcomes

**Figure 5:**
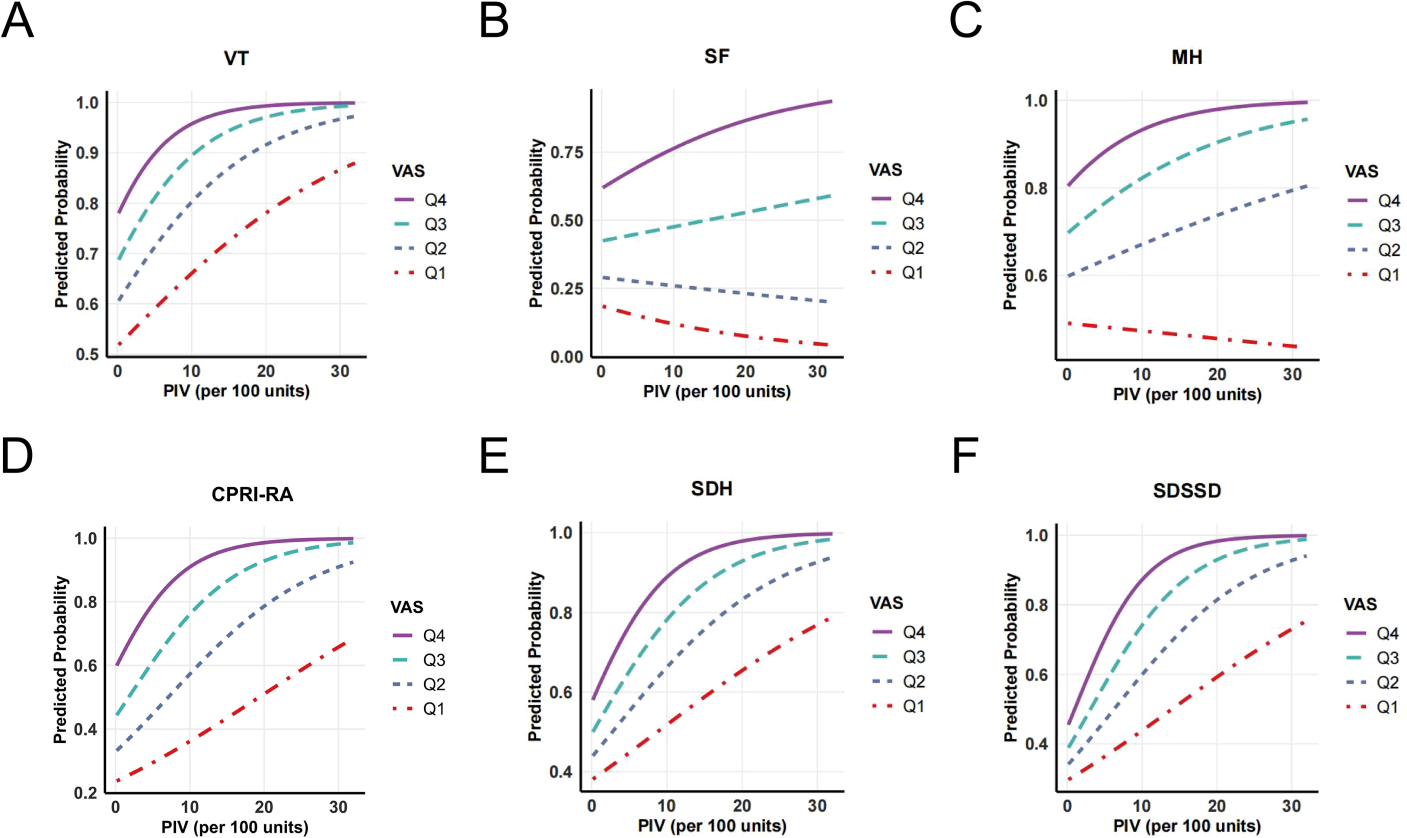
Moderating effects of varying VAS levels on the association between PIV and SPP deterioration risk, including VT **(A)**, SF **(B)**, MH **(C)**, CPRI-RA **(D)**, SDH **(E)**, and SDSSD **(F)**.

**Figure 6:**
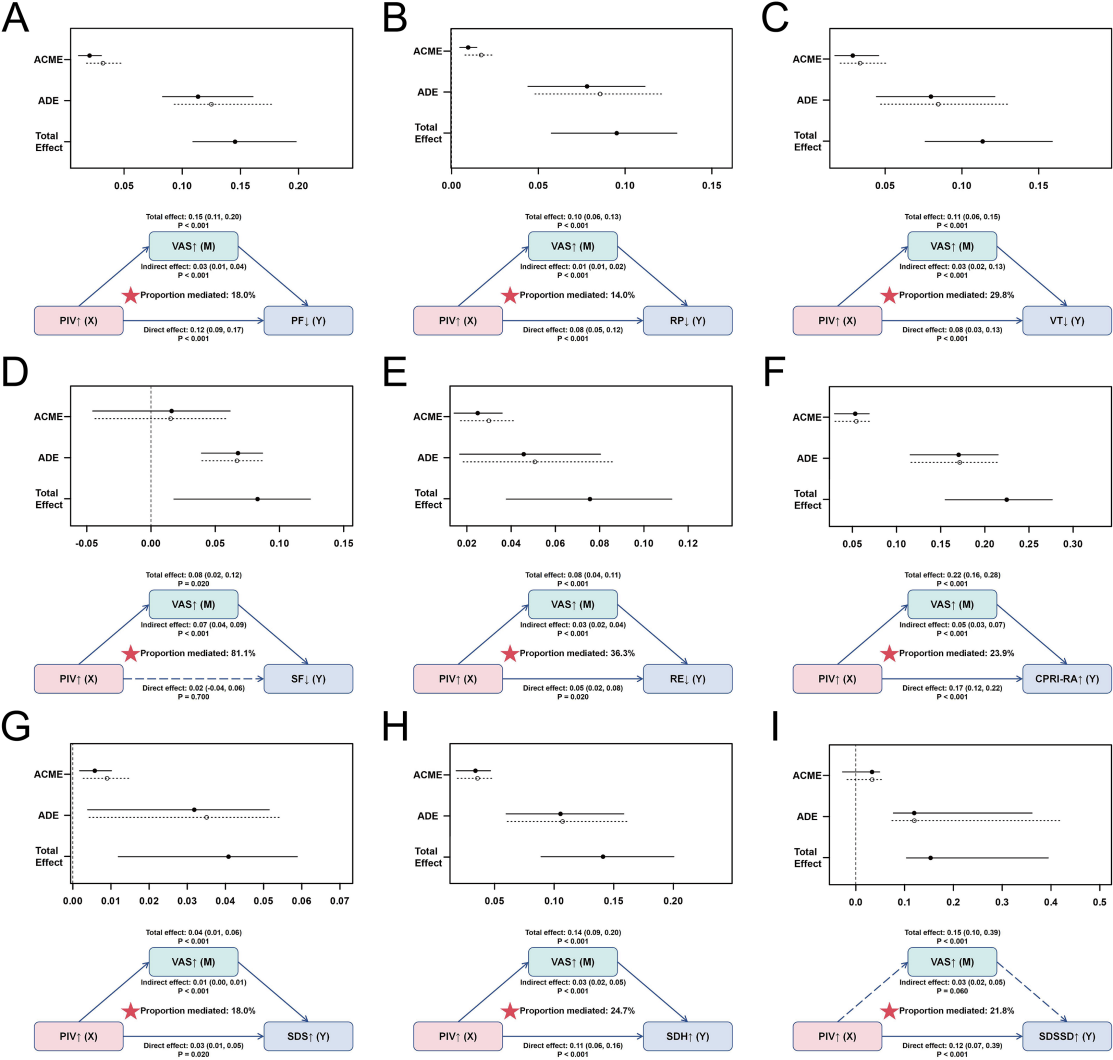
Mediating effects of VAS on the association between PIV and SPP deterioration outcomes, including PF **(A)**, RP **(B)**, VT **(C)**, SF **(D)**, RE **(E)**, CPRI-RA **(F)**, SDS **(G)**, SDH **(H)**, and SDSSD **(I)**.

Given the potential combined effects of PIV and VAS, we further quantified and validated their synergistic associations with SPP deterioration (**Table 4**). After full adjustment for all confounding variables, partial multiplicative interactions were observed between PIV and VAS (P < 0.05). As VAS levels increased, each 100-unit increase in PIV corresponded to a progressively stronger positive association with worsening VT, SF, MH, CPRI-RA, SDH, and SDSSD. Additionally, significant additive interactions between PIV and VAS were detected for the CPRI-RA, SDH, and SDSSD outcomes. Based on RERI values, excess risks of deterioration in CPRI-RA, SDH, and SDSSD among individuals jointly exposed to high PIV and high VAS were 5.44-, 1.61-, and 1.46-fold higher, respectively. The attributable proportion (AP) values indicated that 57%, 38%, and 35% of the risks for these outcomes could be attributed to the PIV–VAS interaction. The SI values further supported the presence of a synergistic interaction between the two parameters. However, for VT, SF, and MH outcomes, additive interactions did not reach statistical significance. Furthermore, moderation analysis was performed to evaluate whether VAS levels moderated the associations between PIV and adverse SPP outcomes (**Figure 5**). A consistent pattern was observed across the simple slope plots for different outcomes, wherein higher VAS scores invariably strengthened the associations between PIV and the risk of deterioration in VT, SF, MH, CPRI-RA, SDH, and SDSSD. This underscores the stable role of VAS as an effect modifier. Additionally, VAS was examined as a potential mediator of the association between PIV and the deterioration of SPP outcomes (**Figure 6**). Mediation analysis revealed that, independent of all confounding variables, VAS partially mediated the association between PIV and the worsening of PF, RP, VT, RE, CPRI-RA, SDS, and SDH outcomes, with indirect effect proportions of 18.0%, 14.0%, 29.8%, 36.3%, 23.9%, 18.0%, and 24.7%, respectively. Interestingly, VAS mediated 81.1% of the association between PIV and SF outcomes, while the direct effect was not statistically significant, suggesting a potential full mediation effect. In contrast, for SDSSD outcomes, the effect of PIV was primarily direct, and the mediating effect of VAS did not reach statistical significance.

**Table 4:** Association between VAS and SPP variance risk.

Outcome	Interactive indices				
	Multiplicative effect		Additive effect		
	β(95%CI)	P value	RERI(95%CI)	AP(95%CI)	SI(95%CI)
VT	0.04(0.01, 0.08)	0.042	1.44(-0.10, 3.38)	0.32(-0.02, 0.55)	1.70(0.97, 3.17)
SF	0.04(0.01, 0.07)	0.014	1.19(-0.87, 3.68)	0.17(-0.14, 0.43)	1.25(0.86, 1.93)
MH	0.04(0.01, 0.08)	0.019	0.88(-0.27, 2.07)	0.28(-0.10, 0.53)	1.69(0.86, 4.18)
CPRI-RA	0.04(0.01, 0.08)	0.023	5.44(3.24, 9.13)	0.57(0.41, 0.70)	2.79(1.89, 4.39)
SDH	0.04(0.01, 0.08)	0.024	1.61(0.41, 3.05)	0.38(0.11, 0.57)	2.00(1.18, 3.89)
SDSSD	0.05(0.02, 0.08)	0.004	1.46(0.38, 2.84)	0.35(0.11, 0.55)	1.88(1.17, 3.38)

### 3.6 Subgroup analysis of the combined effect of PIV and VAS on the risk of SPP deterioration

We performed stratified analyses to evaluate the association between PIV and VAS levels and the risk of SPP deterioration across different participant subgroups (**Supplementary Figure 4**). In most predefined and exploratory subgroups, including gender, age, BMI, disease duration, and CCI, these factors did not significantly modify the relationship between combined PIV–VAS exposure and SPP deterioration risk. Notably, participants in the high PIV and high VAS group consistently exhibited the greatest risk of SPP deterioration. Furthermore, a significant interaction between age and the combined PIV–VAS effect was observed for PF, BP, and CPRI-RA outcomes (interaction P < 0.05). Specifically, the joint impact of elevated PIV and VAS levels on PF, BP, and CPRI-RA deterioration was more pronounced among participants younger than 60 years.

### 3.7 Sensitivity analysis of the combined effect of PIV and VAS on the risk of SPP deterioration

**Figure 7:**
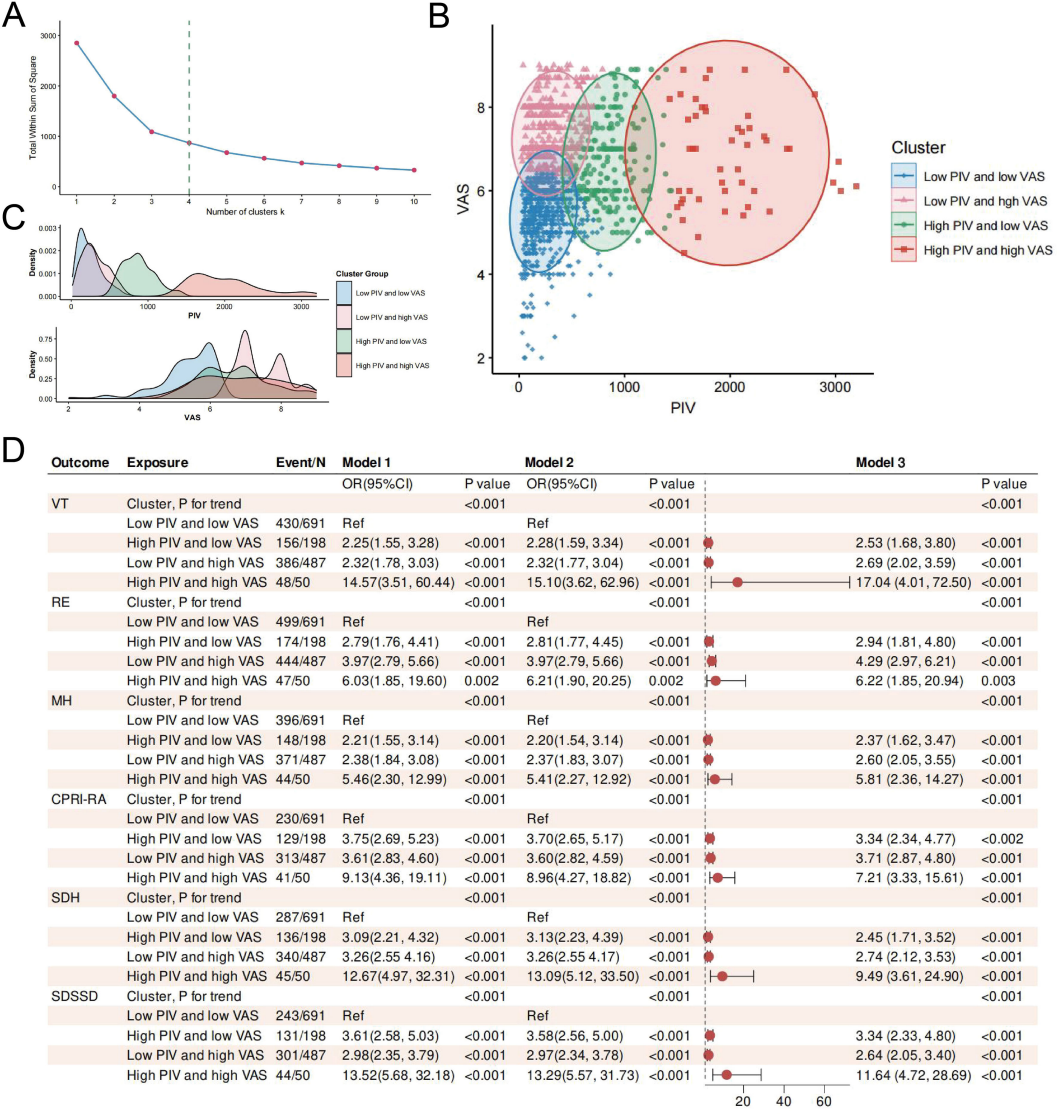
Clustering and regression analyses based on PIV and VAS levels. **(A)** Clustering of PIV and VAS levels using the K-means clustering algorithm. **(B)** Identification of four clusters using the Euclidean distance method. **(C)** Distribution of PIV and VAS values across the four clusters. **(D)** Stepwise-adjusted logistic regression analysis of SPP deterioration risk across different clusters.

To assess the robustness of the study findings, multiple sensitivity analyses were performed. First, k-means clustering was used to reclassify participants into four distinct PIV–VAS subgroups (**Figure 7A**): Group 1 (n = 691), characterized by both low PIV and low VAS levels; Group 2 (n = 198), with low PIV and high VAS levels; Group 3 (n = 487), with high PIV and low VAS levels; and Group 4 (n = 50), with both PIV and VAS at the highest levels (**Figures 7B, C**). After adjustment for confounding variables, participants with high PIV and high VAS levels continued to exhibit the highest risks of deterioration in VT, RE, MH, CPRI-RA, SDH, and SDSSD (**Figure 7D**). Subsequently, ordinal logistic regression analysis further confirmed trends consistent with those of the primary models. Compared with the low PIV and low VAS group, the high PIV and high VAS group continued to demonstrate elevated risks of deterioration in PF, BP, VT, SF, MH, CPRI-RA, SDS, SDH, and SDSSD outcomes, as presented in **Supplementary Table 6**. Furthermore, when GAMs incorporating additional smoothing terms for continuous covariates were applied, the associations between combined PIV–VAS exposure and deterioration in PF, BP, VT, SF, RE, MH, CPRI-RA, SDS, SDH, and SDSSD outcomes remained consistent with the primary findings (**Supplementary Table 7**). Analyses using AIC and BIC further supported the stability and reliability of the fitted models (**Supplementary Table 8**). In addition, E-values were calculated to evaluate the potential influence of unmeasured confounding. All E-values exceeded 5, suggesting that any unknown or unmeasured confounders would need to have a strong association with both the exposure and outcome to fully explain the observed relationships. Collectively, all sensitivity analyses confirmed that the study results were robust.

### 3.8 Superior discriminatory performance of the combined PIV and VAS model in predicting SPP outcomes

**Figure 8:**
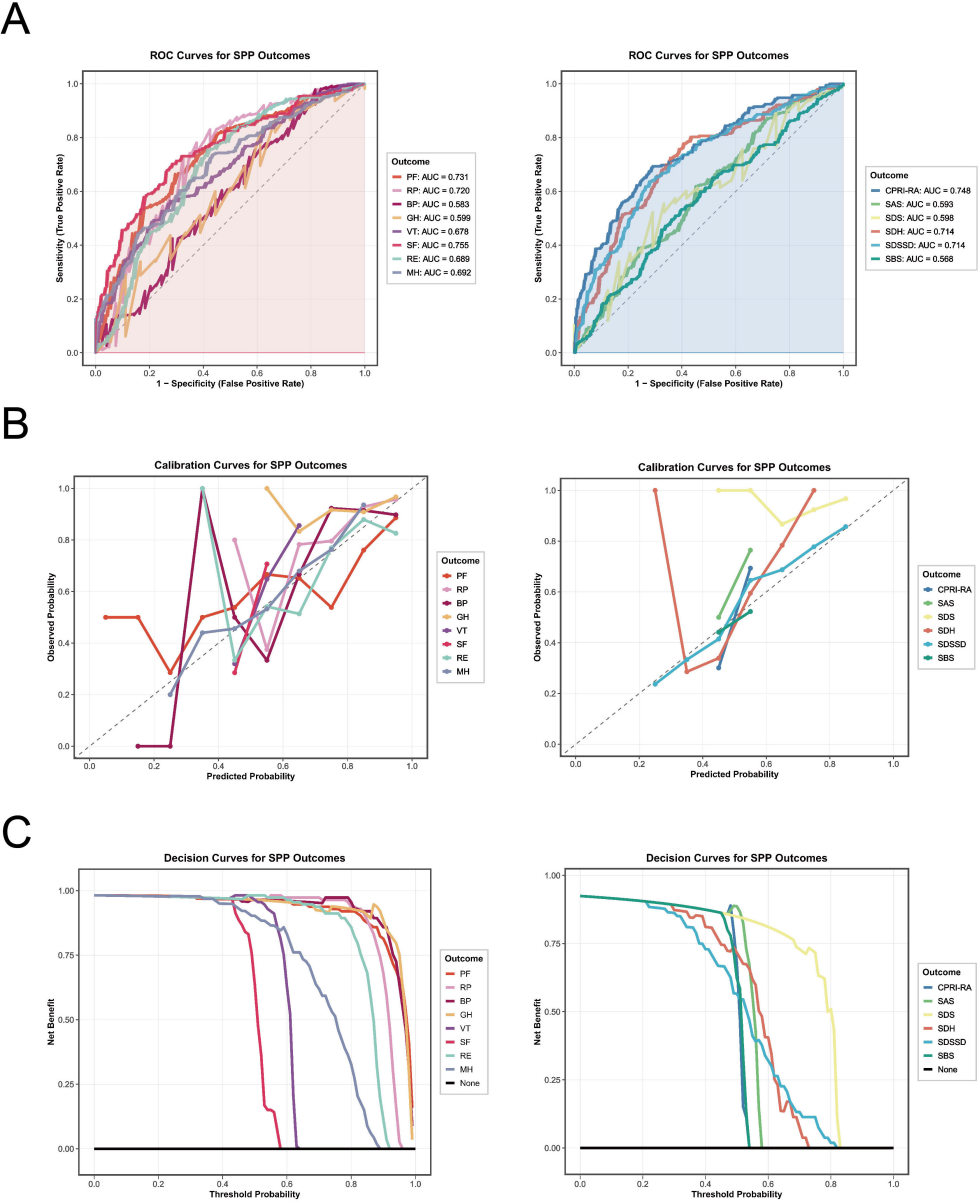
ROC curves **(A)**, calibration plots **(B)**, and DCA **(C)** for the SPP risk prediction models in the testing cohort.

To further evaluate the predictive value of PIV and VAS for SPP deterioration outcomes, a total of 16 feature variables—including PIV and VAS—were selected for model construction. Using random sampling, the entire cohort was divided into a training cohort (n = 998) and a testing cohort (n = 428) at a 7:3 ratio. Descriptive statistics for both cohorts are presented in **Supplementary Table 9**, showing relatively balanced distributions without statistically significant differences. Based on these data, predictive models for 14 SPP deterioration outcomes were developed using the XGBoost algorithm within the training cohort. Following five-fold cross-validation, the optimal combinations of hyperparameters for each of the 14 models were identified, as summarized in **Supplementary Table 10**. The performance metrics of each model are presented in **Table 5**. Among the models, the SF model achieved the highest AUC value (0.755; 95% CI: 0.705–0.800), followed by the CPRI-RA model (0.748; 95% CI: 0.701–0.795), and then the PF and RP models (**Figure 8A**). The GH model demonstrated higher SEN and SPE, at 0.801 and 0.820, respectively. The GH and SDS models yielded the highest PPVs, at 0.981 and 0.974, respectively. In terms of F1-scores, the models showing the best performance were GH, RP, and BP, in that order. The calibration curves and decision curve analyses for all SPP models are displayed in **Figures 8B, C**. Comparatively, the MH and SDSSD models exhibited better consistency between observed and predicted outcomes, while the GH, BP, and PF models demonstrated greater net clinical benefit across most threshold probability ranges.

**Table 5:** Predictive performance metrics of the SPP risk model in the test cohort.

Outcome	AUC (95%CI)	Accuracy	Sensitivity	Specificity	PLR	NLR	PPV	NPV	F1 score
PF	0.731(0.670, 0.788)	0.673	0.665	0.705	2.250	0.476	0.897	0.352	0.764
RP	0.720(0.625, 0.808)	0.766	0.781	0.625	2.083	0.351	0.953	0.227	0.858
BP	0.583(0.489, 0.676)	0.715	0.755	0.408	1.275	0.601	0.908	0.177	0.824
GH	0.599(0.456, 0.739)	0.801	0.820	0.389	1.341	0.464	0.968	0.086	0.888
VT	0.678(0.624, 0.731)	0.612	0.564	0.723	2.036	0.603	0.824	0.420	0.669
SF	0.755(0.705, 0.800)	0.710	0.702	0.716	2.471	0.417	0.622	0.783	0.659
RE	0.689(0.619, 0.751)	0.720	0.755	0.584	1.817	0.419	0.874	0.385	0.810
MH	0.692(0.640, 0.745)	0.678	0.736	0.568	1.701	0.466	0.763	0.532	0.749
CPRI-RA	0.748(0.701, 0.795)	0.701	0.693	0.708	2.377	0.433	0.700	0.702	0.697
SAS	0.593(0.531, 0.653)	0.601	0.619	0.549	1.372	0.694	0.793	0.341	0.695
SDS	0.598(0.487, 0.706)	0.533	0.522	0.708	1.791	0.674	0.968	0.081	0.679
SDH	0.714(0.664, 0.763)	0.699	0.778	0.595	1.919	0.374	0.716	0.671	0.746
SDSSD	0.714(0.664, 0.762)	0.678	0.636	0.721	2.282	0.504	0.707	0.652	0.670
SBS	0.568(0.513, 0.624)	0.568	0.651	0.486	1.267	0.718	0.554	0.587	0.599

**Figure 9:**
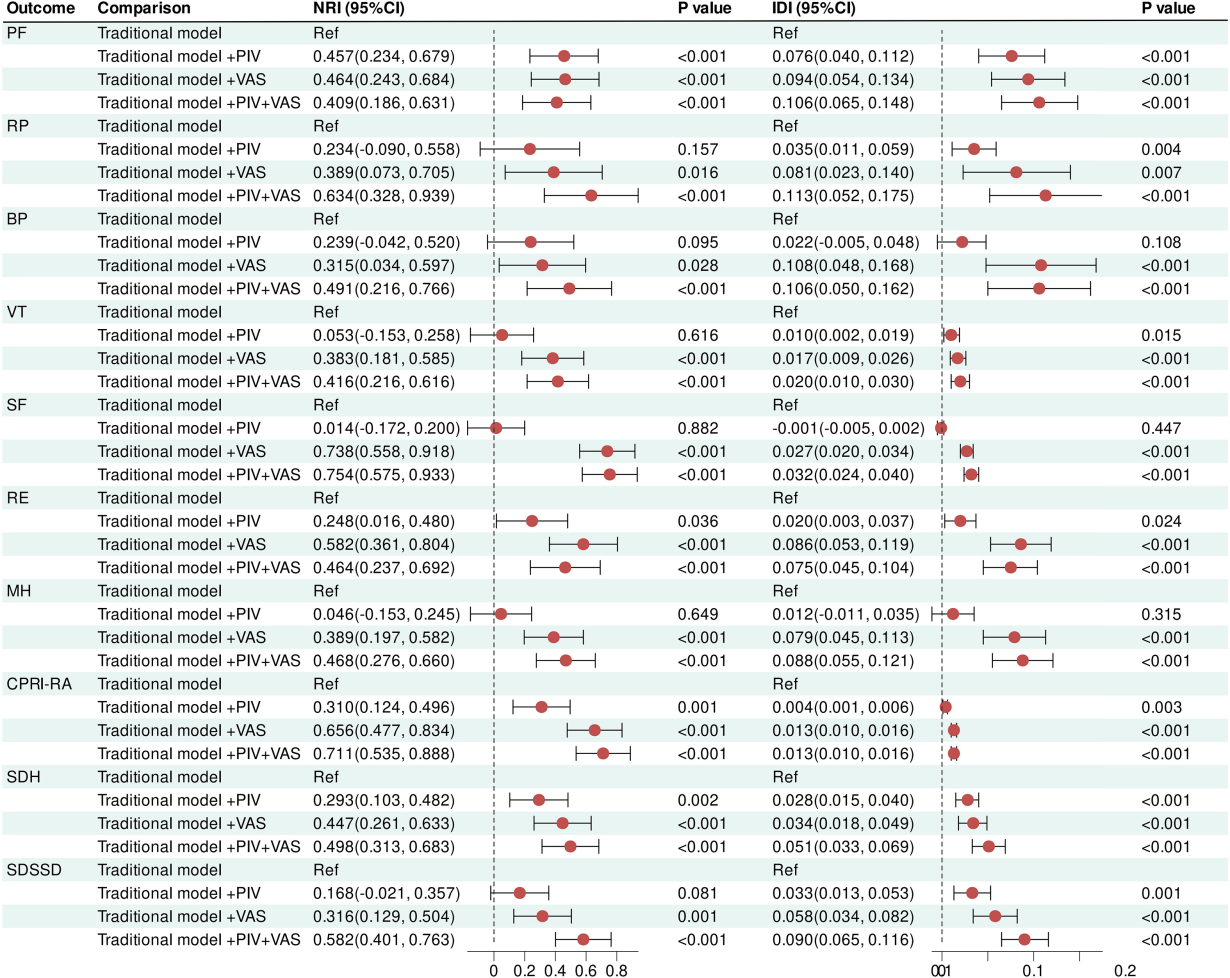
Incremental predictive value of the combined PIV–VAS model compared with traditional risk models.

Additionally, we further evaluated the incremental predictive value of PIV and/or VAS relative to traditional risk factor models (**Figure 9**). Compared with traditional models, the PIV-based model exhibited superior overall predictive performance for PF, RE, CPRI-RA, and SDH outcomes, and significantly enhanced overall discriminatory ability for RP, VT, and SDSSD deterioration outcomes. Notably, the combined PIV–VAS model demonstrated the best overall predictive capacity. Specifically, it yielded the greatest improvement in predicting RP, VT, SF, MH, CPRI-RA, SDH, and SDSSD deterioration outcomes, showing the most pronounced enhancement in overall discriminative ability for PF deterioration and improved classification accuracy for BP deterioration.

### 3.9 Interpretability of PIV and VAS in the optimal outcome model based on SHAP analysis

**Figure 10:**
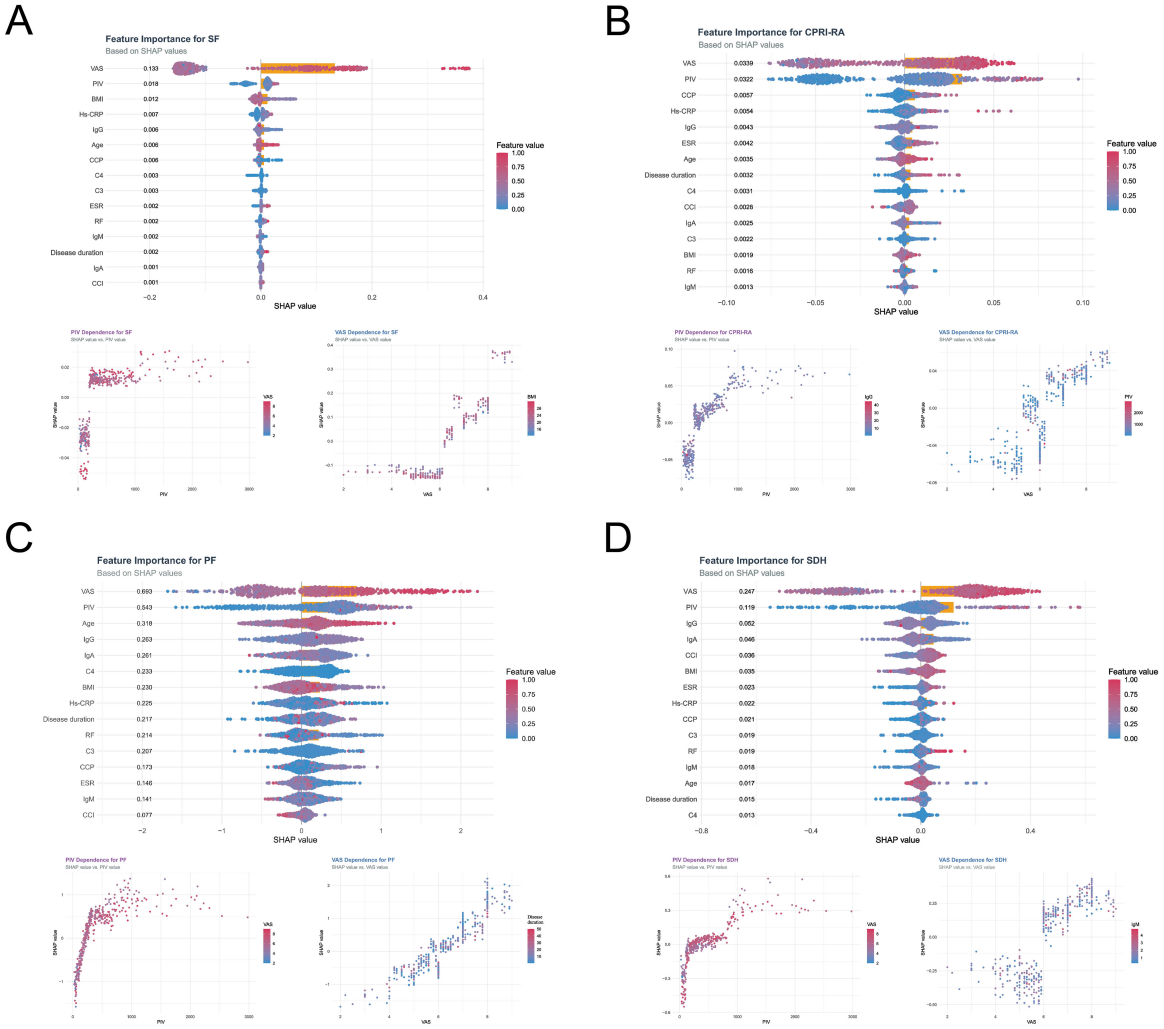
Model interpretability of PIV and VAS based on SHAP analysis in the optimal outcome models for SF **(A)**, CPRI-RA **(B)**, PF **(C)**, and SDH **(D)** outcomes.

To elucidate the mechanisms underlying the complex optimal outcome model, SHAP analysis was applied to illustrate how predictive features influenced adverse outcomes, including SF, CPRI-RA, PF, and SDH. The feature importance ranking and corresponding interpretability of the model are presented in **Figure 10**. Across all models, VAS was identified as the most influential predictor, followed by PIV. Participants with higher VAS and PIV levels (indicated in red) were more likely to experience deterioration in SF, CPRI-RA, PF, and SDH outcomes, as shown on the right side of the SHAP summary plots. SHAP dependency plots were used to visualize how variations in PIV and VAS affected predicted probabilities and to explore potential interactions between features. Individuals with higher PIV and VAS values exhibited higher SHAP scores, indicating an increased risk of deterioration. Furthermore, notable interaction patterns were observed between PIV and VAS. Additional interactions were detected between VAS and BMI, PIV and IgG, VAS and disease duration, and VAS and IgM, suggesting multifactorial associations influencing model predictions.

## 4 Discussion

This study is the first to systematically investigate the combined effects of PIV and VAS on multidimensional composite SPP outcomes in individuals with RA. By integrating conventional statistical analyses with interpretable machine learning algorithms, several key findings were obtained: (1) Elevated PIV and VAS levels were significantly associated with an increased risk of SPP deterioration; (2) Concurrent increases in PIV and VAS exhibited a strong positive association with the likelihood of SPP deterioration; (3) Both multiplicative and additive interactions were observed between PIV and VAS, with VAS acting as both an amplifier of the PIV–SPP association and a mediating factor; and (4) The combined PIV–VAS model markedly improved the predictive performance for SPP outcomes, identifying VAS and PIV as the most influential predictors.

PIV is a comprehensive multi-immune biomarker, the main advantage of which lies in its ability to integrate multiple hematologic parameters and reflect dynamic changes in systemic inflammatory responses across several dimensions (35). Our study confirmed an independent positive correlation and nonlinear association between PIV and the risk of SPP deterioration, encompassing multiple domains such as PF, RP, BP, VT, SF, RECPRI-RA, SDH, and SDSSD. The interpretable machine learning model further identified PIV as a major predictive feature for SPP deterioration, demonstrating significantly greater importance than traditional inflammatory markers such as ESR and CRP. These findings indicate that PIV overcomes the limitations of conventional single biomarkers by providing a more integrated and nuanced assessment of systemic inflammatory burden. Mechanistically, elevated PIV levels are closely linked to the excessive release of inflammatory mediators, immune cell dysregulation, and progressive tissue damage (36). When tissue injury occurs, neutrophils rapidly secrete chemokines, reactive oxygen species, and neutrophil extracellular traps, which infiltrate and amplify inflammation within the synovial compartment (37). Chronic inflammatory states are frequently accompanied by lymphopenia, which may result from increased lymphocyte apoptosis, impaired proliferation and differentiation, and redistribution within lymphoid tissues (38). Monocytes serve as circulating precursors of macrophages and osteoclasts, thereby exacerbating synovial inflammation and promoting bone erosion in RA (39). In addition, activated platelets release pro-inflammatory cytokines such as interleukin-1β and transforming growth factor-β, as well as angiogenic factors that directly stimulate synovial neovascularization and contribute to joint structural damage (40). These immunopathological mechanisms are believed to play a pivotal role in driving the multidimensional manifestations of RA. Mardan et al. (41) recently conducted a meta-analysis demonstrating that PIV is an independent and significant predictor of all-cause mortality in RA, characterized by a nonlinear relationship and a distinct threshold effect. Similarly, Yu et al. (42) reported that elevated PIV levels in patients with gastrointestinal cancers were consistently associated with poorer overall survival and unfavorable clinical outcomes. These findings collectively reinforce the broader applicability of PIV across various disease contexts. In contrast, the principal innovation of the present study lies in extending the application of PIV from acute disease outcomes (such as cancer progression or mortality) to the evaluation of multidimensional clinical outcomes in RA, thereby providing a foundation for its potential utility in the long-term management of chronic inflammatory diseases.

VAS is the most representative and critical tool for evaluating physical pain in patients with RA. Our study confirmed that the VAS demonstrated the broadest and strongest correlations, as well as the highest predictive capacity, for multiple dimensions of SPP outcomes. From a mechanistic perspective, pain in RA primarily arises from nociceptive processes triggered by synovitis and structural joint damage, such as osteoclast-mediated bone erosion (43). This pain can be further exacerbated by central sensitization, characterized by enhanced transmission and amplification of nociceptive signals within the spinal cord (44). Our findings indicate that VAS, as a core indicator of pain intensity, contributes to SPP deterioration through several interrelated pathways. First, physical functional limitation—each 1 cm increase in VAS was associated with an 88% higher risk of PF deterioration, likely due to pain-induced motor inhibition and reduced joint mobility. Second, psychological distress amplification—each 1 cm increase in VAS approximately doubled the risk of deterioration in RE and MH domains, reflecting the bidirectional relationship between chronic pain and emotional dysregulation. Third, social participation restriction—SHAP analysis identified VAS as a dominant predictor in the SF model, underscoring its impact on patients’ engagement in daily and social activities. In summary, these findings emphasize the central role of VAS within the multidimensional clinical outcome framework of RA and highlight the necessity of maintaining adequate pain control below a clinically meaningful threshold to prevent functional and psychosocial deterioration.

Given the observed associations between PIV and VAS in SPP outcomes, statistical models were developed to examine the complex interactions—including interaction, moderation, and mediation effects—in the progression of SPP deterioration. We found that the interactive effect between PIV and VAS exhibited greater statistical significance in predicting the risk of deterioration in CPRI-RA, SDH, and SDSSD. Moreover, as VAS levels increased, the positive association between PIV and adverse SPP outcomes became more pronounced. These findings indicate that maintaining lower pain levels in individuals with heightened inflammatory activity may effectively mitigate the risk of SPP deterioration. Furthermore, mediation analysis confirmed that VAS partially mediated the relationship between PIV and worsening outcomes in physical function, vitality, psychological well-being, and disease activity, highlighting the pivotal role of pain as a mechanistic link between systemic inflammation and multidimensional health impairments in RA. These findings suggest that PIV, which integrates multiple immune cell components, more accurately captures the biological essence of the inflammation–pain interaction. In this framework, VAS functions both as a modulatory factor of PIV and as a conceptual “bridge” linking objective inflammation with subjective pain perception. Simon et al. (45) systematically reported that the Janus kinase/signal transducer and activator of transcription pathway regulates both inflammatory and non-inflammatory pain by modulating the expression and activity of various pro-inflammatory and anti-inflammatory cytokines. Similarly, Krishna et al. (46) proposed that high-mobility group box 1 promotes peripheral and central pain sensitization by activating the Toll-like receptor 4/mitogen-activated protein kinase/nuclear factor κB signaling cascade through its binding to Toll-like receptors and the receptor for advanced glycation end products. These mechanistic insights provide potential biological explanations for the inflammation–pain dual-drive model proposed in the present study.

The integrated assessment of PIV and VAS may provide novel insights with direct implications for the clinical management of RA. Interestingly, for representative CPRI-RA outcomes and TCM-specific endpoints SDH and SDSSD, the combination of PIV and VAS demonstrated high incremental predictive value and significance. Existing literature provides mechanistic support for this integrated approach. Previous studies have shown that active RA typically exhibits intense inflammatory responses, such as markedly elevated serum IL-6 and TNF-α, with activation of TRPV1 channels enhancing pain transmission (47–49). Furthermore, both clinical data from RA patients and experimental rat models have demonstrated consistent associations between spleen deficiency with dampness-heat syndrome and systemic inflammation, as well as significant positive correlations with VAS scores (50–52). Building on this evidence, the predictive model developed in our study offers directly applicable insights for RA management. Primarily, in the context of risk stratification and early identification, the PIV-VAS combination constitutes an easily accessible and cost-effective tool for efficient screening of patients at high risk for SPP deterioration, enabling clinicians to rapidly identify candidates requiring prioritized intervention during routine clinical practice. Secondly, regarding dynamic monitoring and personalized treatment strategies, the “inflammation-pain dual-driver model” proposed in our study reveals that clinical interventions should extend beyond unidimensional approaches. In addition to controlling systemic immune-inflammatory responses, it is essential to integrate effective multimodal strategies incorporating patient self-management education and cognitive-behavioral therapy. Subsequent research should focus on developing clinical decision support systems that incorporate both PIV and VAS into standard RA follow-up protocols and prospectively validate the efficacy of personalized intervention strategies based on this model for improving long-term patient outcomes and treatment adherence.

This pioneering work offers several notable advantages. First, it innovatively integrates findings from international consensus reports with elements of TCM syndrome differentiation, thereby overcoming the limitations of single-outcome assessments. This integration establishes a composite, multidimensional outcome evaluation framework that reflects an in-depth exploration of the synergy between traditional Chinese and Western medicine. Second, it introduces the novel “inflammation–pain dual-driver model,” which combines the PIV—a newly recognized objective biomarker of inflammation—with VAS—a classical subjective measure of pain—to construct a synergistic analytical framework. This approach moves beyond the traditional single-biomarker research paradigm by emphasizing the interactive effects of multidimensional indicators. Third, this study cross-validates traditional statistical analyses with interpretable machine learning algorithms, thereby addressing the inherent limitations of conventional “black-box” models. This integration enables quantitative interpretation of the interaction effects and predictive contributions of combined indicators, demonstrating the methodological rigor of a multi-model, multi-algorithm analytical strategy. However, several limitations should be acknowledged. Foremost among these is the retrospective design. Although we employed multivariable adjustment and extensive sensitivity analyses to minimize confounding, the possibility of residual confounding cannot be entirely ruled out. Unmeasured factors such as specific medication regimens could potentially influence the outcomes. To address this, we calculated E-values, which indicated that an unmeasured confounder of considerable strength would be needed to explain away our primary associations, lending support to the robustness of our findings. Secondly, as an emerging biomarker, PIV lacks universally established cut-off values. To mitigate this, we applied K-means clustering in sensitivity analyses to reclassify PIV and VAS status, yielding results consistent with the main analysis. Third, the single-center design may limit the generalizability of our predictive model. While we assessed internal stability via bootstrap resampling and confirmed feature effect consistency using SHAP dependence plots, future external validation in prospective, multi-center cohorts is necessary to confirm its broad applicability.

## 5 Conclusion

Our findings, validated through both traditional statistical analyses and interpretable machine learning approaches, demonstrate that elevated PIV and VAS levels independently and synergistically increase the risk of SPP deterioration in RA. The interaction between PIV and VAS substantially amplifies the likelihood of adverse multidimensional outcomes, with VAS acting as a key mediating pathway through which PIV influences SPP deterioration. A predictive model integrating PIV and VAS effectively identifies individuals at high risk for SPP worsening, underscoring the clinical importance of their combined assessment in stratifying and screening high-risk RA populations. Collectively, these findings provide a novel framework for managing the inflammation–pain axis in RA and highlight the potential of PIV–VAS integration as an accessible and practical tool for individualized patient monitoring and clinical decision-making.

## Data Availability

The original contributions presented in the study are included in the article/**Supplementary Material**. Further inquiries can be directed to the corresponding author.
