# Supplementary material for: Joint association between pan-immune inflammation value and physical pain with self-perception of patients in rheumatoid arthritis: a retrospective cohort study employing traditional statistics and interpretable machine learning
Source: Front Immunol. 2025 Dec 18;16:1711081. doi: 10.3389/fimmu.2025.1711081 (PMC12756397; doi:10.3389/fimmu.2025.1711081)
Supplement: Supplementary file 1 [file Table1.docx]

## Supplementary Tables

**Supplementary Table 1 The proportion of missing covariates and imputation methods**

| **Variable** | **Number of missing data** | **Percentage of missing data (%)** | **Imputation methods** |
| --- | --- | --- | --- |
| ESR | 14 | 0.98 | logreg |
| Hs-CRP | 9 | 0.63 | logreg |
| RF | 5 | 0.35 | logreg |
| CCP | 42 | 2.95 | logreg |
| IgA | 34 | 2.38 | logreg |
| IgG | 34 | 2.38 | logreg |
| IgM | 34 | 2.38 | logreg |
| C3 | 36 | 2.52 | logreg |
| C4 | 36 | 2.52 | logreg |

**Supplementary Table 2 Baseline characteristics stratified by the quartiles of PIV**

| **Characteristic** | **Quartiles of PIV** | | | | **P value** |
| --- | --- | --- | --- | --- | --- |
|  | **Q1  (≤161.19)** | **Q2  (161.19-288.95)** | **Q3  (288.95-522.14)** | **Q4  (>522.14)** |  |
| **Participants** | 356 | 357 | 357 | 356 |  |
| **Gender, n (%)** |  |  |  |  | <0.001 |
| Male | 37 (10.4%) | 58 (16.2%) | 68 (19.0%) | 88 (24.7%) |  |
| Female | 319 (89.6%) | 299 (83.8%) | 289 (81.0%) | 268 (75.3%) |  |
| **Age (years)** | 57.0 (51.0, 67.0) | 58.0 (51.0, 68.0) | 59.0 (51.5, 69.0) | 60.0 (53.0, 69.0) | 0.012 |
| **Age (years), n (%)** |  |  |  |  | 0.010 |
| <60 | 216 (60.7%) | 206 (57.7%) | 192 (53.8%) | 174 (48.9%) |  |
| ≥60 | 140 (39.3%) | 151 (42.3%) | 165 (46.2%) | 182 (51.1%) |  |
| **BMI (kg/m^2^)** | 22.25 (20.40, 24.07) | 22.14 (20.31, 23.63) | 22.32 (20.20, 24.38) | 22.11 (20.23, 23.58) | 0.369 |
| **Disease duration (years)** | 10.0 (4.4, 17.0) | 8.3 (3.1, 15.0) | 8.3 (3.0, 16.0) | 9.0 (4.0, 15.0) | 0.072 |
| **CCI (score)** | 4.0 (3.0, 5.8) | 4.0 (3.0, 6.0) | 4.0 (3.0, 6.0) | 5.0 (4.0, 6.0) | 0.068 |
| **ESR (mm/h)** | 22.00 (10.00, 40.00) | 32.00 (16.00, 50.50) | 38.00 (21.00, 57.50) | 48.50 (31.00, 73.00) | <0.001 |
| **Hs-CRP (mg/L)** | 3.46 (1.26, 10.45) | 9.32 (2.84, 23.94) | 17.82 (6.28, 38.35) | 34.09 (11.93, 67.49) | <0.001 |
| **RF (KIU/L)** | 76.30 (34.00, 201.13) | 88.50 (31.40, 235.40) | 118.00 (48.30, 285.30) | 123.95 (52.75, 346.28) | <0.001 |
| **CCP (U/ml)** | 96.95 (11.65, 236.99) | 101.00 (18.95, 273.50) | 81.30 (16.25, 241.50) | 79.40 (16.13, 239.00) | 0.683 |
| **IgA (g/L)** | 2.63 (1.93, 3.60) | 2.76 (2.04, 3.63) | 2.88 (2.09, 3.75) | 2.95 (2.16, 4.04) | 0.018 |
| **IgG (g/L)** | 11.75 (9.53, 15.01) | 11.74 (9.68, 14.41) | 11.54 (9.40, 14.24) | 11.13 (9.15, 14.05) | 0.177 |
| **IgM (g/L)** | 1.20 (0.87, 1.73) | 1.32 (0.95, 1.83) | 1.27 (0.90, 1.71) | 1.24 (0.94, 1.69) | 0.526 |
| **C3 (g/L)** | 1.12 (0.99, 1.27) | 1.19 (1.07, 1.34) | 1.26 (1.14, 1.41) | 1.30 (1.15, 1.46) | <0.001 |
| **C4 (g/L)** | 0.27 (0.21, 0.33) | 0.30 (0.24, 0.36) | 0.32 (0.25, 0.39) | 0.31 (0.24, 0.39) | <0.001 |
| **PIV** | 106.19 (70.40, 131.52) | 226.03 (192.58, 259.24) | 378.63 (327.58, 449.96) | 793.81 (624.47, 1,078.07) | <0.001 |
| **VAS (cm)** | 5.70 (5.00, 6.78) | 6.20 (5.60, 7.00) | 6.40 (5.70, 7.00) | 6.95 (5.80, 7.68) | <0.001 |
| **PF (score)** | 35.00 (30.00, 55.00) | 30.00 (25.00, 45.00) | 30.00 (20.00, 35.00) | 30.00 (20.00, 35.00) | <0.001 |
| **RP (score)** | 0.00 (0.00, 25.00) | 0.00 (0.00, 25.00) | 0.00 (0.00, 25.00) | 0.00 (0.00, 25.00) | <0.001 |
| **BP (score)** | 41.00 (30.99, 41.00) | 31.00 (22.00, 41.00) | 31.00 (22.00, 41.00) | 31.00 (22.00, 41.00) | <0.001 |
| **GH (score)** | 30.00 (25.00, 40.00) | 30.00 (20.00, 35.00) | 30.00 (20.00, 35.00) | 25.00 (20.00, 35.00) | <0.001 |
| **VT (score)** | 45.00 (35.00, 51.50) | 40.00 (30.00, 50.00) | 40.00 (30.00, 50.00) | 35.00 (25.00, 45.00) | <0.001 |
| **SF (score)** | 50.00 (37.50, 62.50) | 50.00 (37.50, 62.50) | 50.00 (37.50, 62.50) | 37.50 (25.00, 50.00) | <0.001 |
| **RE (score)** | 33.33 (0.00, 66.66) | 33.33 (0.00, 33.33) | 33.33 (0.00, 33.33) | 0.00 (0.00, 33.33) | <0.001 |
| **MH (score)** | 48.00 (36.00, 56.00) | 44.00 (36.00, 52.00) | 44.00 (36.00, 52.00) | 40.00 (28.50, 50.00) | <0.001 |
| **CPRI-RA (score)** | 8.80 (7.70, 10.24) | 9.61 (8.29, 10.96) | 10.01 (8.77, 11.22) | 10.60 (9.28, 11.77) | <0.001 |
| **SAS (score)** | 52.50 (47.50, 56.25) | 53.75 (50.00, 58.75) | 53.75 (50.00, 58.75) | 53.75 (48.75, 58.75) | 0.002 |
| **SDS (score)** | 59.38 (55.00, 63.75) | 60.00 (56.25, 66.25) | 61.25 (57.50, 66.88) | 62.50 (57.50, 70.00) | <0.001 |
| **SDH (score)** | 14 (11, 17.0) | 15.0 (12.0, 19.0) | 16.0 (13.0, 19.0) | 17.0 (14.0, 19.8) | <0.001 |
| **SDSSD (score)** | 11.0 (8.0, 16.0) | 13.0 (10.0, 18.0) | 14.0 (10.0, 18.0) | 16.0 (12.0, 19.0) | <0.001 |
| **SBS (score)** | 6.0 (4.0, 8.0) | 5.0 (4.0, 8.0) | 6.0 (4.0, 7.0) | 6.0 (4.0, 8.0) | 0.415 |

Categorical variables were shown as n (percent, %). Continuous variables were shown as median (Interquartile range, IQR). *P* values for differences between groups were derived using a Pearson’s Chi-squared test or Kruskal-Wallis rank sum test.

**Supplementary Table 3 Baseline characteristics stratified by the quartiles of VAS**

| **Characteristic** | **Quartiles of VAS** | | | | **P value** |
| --- | --- | --- | --- | --- | --- |
|  | **Q1  (≤161.19)** | **Q2  (161.19-288.95)** | **Q3  (288.95-522.14)** | **Q4  (≥522.14)** |  |
| **Participants** | 380 | 372 | 349 | 325 |  |
| **Gender, n (%)** |  |  |  |  | 0.388 |
| Male | 64 (16.8%) | 57 (15.3%) | 65 (18.6%) | 65 (20.0%) |  |
| Female | 316 (83.2%) | 315 (84.7%) | 284 (81.4%) | 260 (80.0%) |  |
| **Age (years)** | 57.0 (50.3, 68.0) | 58.0 (52.0, 68.0) | 58.0 (50.0, 68.0) | 60.0 (52.0, 69.0) | 0.135 |
| **Age (years), n (%)** |  |  |  |  | 0.039 |
| <60 | 223 (58.7%) | 214 (57.5%) | 193 (55.3%) | 158 (48.6%) |  |
| ≥60 | 157 (41.3%) | 158 (42.5%) | 156 (44.7%) | 167 (51.4%) |  |
| **BMI (kg/m^2^)** | 22.21 (20.41, 24.03) | 22.15 (19.99, 23.94) | 22.40 (20.57, 24.22) | 21.93 (20.20, 23.42) | 0.078 |
| **Disease duration (years)** | 9.0 (3.0, 16.0) | 8.0 (3.0, 15.0) | 10.0 (3.0, 16.9) | 10.0 (5.1, 16.0) | 0.025 |
| **CCI (score)** | 4.0 (3.0, 5.0) | 4.0 (3.0, 5.0) | 5.0 (3.0, 6.0) | 5.0 (4.0, 6.0) | <0.001 |
| **ESR (mm/h)** | 29.00 (14.00, 49.75) | 34.00 (15.00, 54.00) | 36.00 (19.00, 54.50) | 45.00 (23.50, 69.00) | <0.001 |
| **Hs-CRP (mg/L)** | 7.03 (2.18, 26.47) | 11.93 (3.17, 33.66) | 13.03 (4.01, 35.66) | 19.95 (5.61, 46.84) | <0.001 |
| **RF (KIU/L)** | 95.40 (38.85, 239.85) | 106.35 (34.13, 256.35) | 88.80 (35.70, 238.95) | 146.80 (47.70, 309.00) | 0.009 |
| **CCP (U/ml)** | 100.00 (14.25, 275.27) | 75.75 (19.00, 231.75) | 92.60 (11.15, 267.50) | 90.50 (21.50, 216.00) | 0.776 |
| **IgA (g/L)** | 2.74 (1.99, 3.77) | 2.84 (2.04, 3.75) | 2.74 (2.05, 3.57) | 2.94 (2.19, 3.97) | 0.106 |
| **IgG (g/L)** | 11.99 (10.01, 15.07) | 11.47 (9.50, 14.22) | 11.18 (9.12, 14.18) | 11.51 (9.16, 14.34) | 0.006 |
| **IgM (g/L)** | 1.24 (0.92, 1.75) | 1.23 (0.95, 1.71) | 1.27 (0.88, 1.8) | 1.33 (0.89, 1.83) | 0.794 |
| **C3 (g/L)** | 1.21 (1.05, 1.36) | 1.22 (1.10, 1.37) | 1.23 (1.10, 1.37) | 1.22 (1.07, 1.37) | 0.593 |
| **C4 (g/L)** | 0.29 (0.22, 0.37) | 0.31 (0.24, 0.37) | 0.31 (0.24, 0.38) | 0.29 (0.24, 0.37) | 0.324 |
| **PIV** | 198.55 (107.82, 366.17) | 274.03 (163.14, 482.67) | 322.94 (201.18, 520.70) | 399.17 (222.85, 693.67) | <0.001 |
| **VAS (cm)** | 5.00 (4.60, 5.30) | 6.00 (5.80, 6.00) | 7.00 (6.50, 7.00) | 8.00 (7.60, 8.20) | <0.001 |
| **PF (score)** | 35.00 (30.00, 50.00) | 35.00 (25.00, 45.00) | 30.00 (20.00, 35.00) | 25.00 (20.00, 30.00) | <0.001 |
| **RP (score)** | 0.00 (0.00, 25.00) | 22.50 (0.00, 25.00) | 0.00 (0.00, 25.00) | 0.00 (0.00, 0.00) | <0.001 |
| **BP (score)** | 41.00 (31.00, 41.00) | 32.00 (30.99, 41.00) | 31.00 (22.00, 41.00) | 22.00 (22.00, 32.00) | <0.001 |
| **GH (score)** | 30.00 (25.00, 40.00) | 30.00 (25.00, 35.00) | 30.00 (20.00, 35.00) | 20.00 (15.00, 30.00) | <0.001 |
| **VT (score)** | 45.00 (40.00, 55.00) | 40.00 (35.00, 50.00) | 40.00 (30.00, 45.00) | 30.00 (20.00, 40.00) | <0.001 |
| **SF (score)** | 50.00 (50.00, 62.50) | 50.00 (50.00, 62.50) | 50.00 (31.25, 50.00) | 25.00 (25.00, 50.00) | <0.001 |
| **RE (score)** | 33.33 (0.00, 66.66) | 33.33 (0.00, 33.33) | 33.33 (0.00, 33.33) | 0.00 (0.00, 33.33) | <0.001 |
| **MH (score)** | 48.00 (44.00, 56.00) | 44.00 (36.00, 52.00) | 44.00 (32.00, 52.00) | 32.00 (20.00, 44.00) | <0.001 |
| **CPRI-RA (score)** | 8.67 (7.62, 10.03) | 9.18 (8.16, 10.33) | 10.33 (9.22, 11.24) | 11.42 (9.89, 13.03) | <0.001 |
| **SAS (score)** | 52.50 (48.75, 55.00) | 53.75 (50.00, 56.25) | 53.75 (46.88, 58.75) | 56.25 (48.75, 65.00) | <0.001 |
| **SDS (score)** | 58.75 (56.25, 62.50) | 58.75 (55.00, 62.50) | 61.25 (56.88, 66.25) | 68.75 (61.25, 78.12) | <0.001 |
| **SDH (score)** | 13.5 (11.0, 16.0) | 14.0 (12.0, 18.0) | 17.0 (13.0, 20.5) | 17.0 (15.0, 21.0) | <0.001 |
| **SDSSD (score)** | 11.0 (8.0, 14.0) | 12.5 (10.0, 16.0) | 15.0 (10.5, 19.0) | 17.0 (13.0, 20.0) | <0.001 |
| **SBS (score)** | 5.0 (4.0, 7.0) | 6.0 (4.0, 7.0) | 6.0 (4.0, 8.0) | 5.0 (4.0, 8.0) | 0.052 |

Categorical variables were shown as n (percent, %). Continuous variables were shown as median (Interquartile range, IQR). *P* values for differences between groups were derived using a Pearson’s Chi-squared test or Kruskal-Wallis rank sum test.

**Supplementary Table 4 Correlation between PIV, VAS and laboratory indicators**

| **Variables** | **PIV** | | **VAS** | |
| --- | --- | --- | --- | --- |
|  | **r** | **p** | **r** | **p** |
| **ESR** | 0.335 | <0.001 | 0.186 | <0.001 |
| **Hs-CRP** | 0.476 | <0.001 | 0.169 | <0.001 |
| **RF** | 0.129 | <0.001 | 0.051 | 0.056 |
| **CCP** | 0.002 | 0.934 | -0.003 | 0.915 |
| **IgA** | 0.087 | 0.001 | 0.030 | 0.251 |
| **IgG** | -0.054 | 0.043 | -0.091 | 0.001 |
| **IgM** | 0.019 | 0.471 | 0.020 | 0.457 |
| **C3** | 0.292 | <0.001 | 0.008 | 0.752 |
| **C4** | 0.149 | <0.001 | 0.022 | 0.412 |

**Supplementary Table 5 Correlation between baseline characteristics and clinical variables**

| **Variables** | **Age** | | **BMI** | | **Disease duration** | | **CCI** | |
| --- | --- | --- | --- | --- | --- | --- | --- | --- |
|  | **r** | **p** | **r** | **p** | **r** | **p** | **r** | **p** |
| **PIV** | 0.089 | <0.001 | -0.022 | 0.396 | -0.056 | 0.035 | 0.061 | 0.021 |
| **VAS** | 0.042 | 0.113 | -0.019 | 0.484 | 0.047 | 0.075 | 0.186 | <0.001 |
| **ESR** | 0.117 | <0.001 | -0.108 | <0.001 | -0.025 | 0.348 | 0.125 | <0.001 |
| **Hs-CRP** | 0.141 | <0.001 | -0.068 | 0.01 | -0.006 | 0.822 | 0.083 | 0.002 |
| **RF** | 0.003 | 0.897 | -0.016 | 0.543 | -0.033 | 0.216 | -0.026 | 0.321 |
| **CCP** | 0.001 | 0.968 | 0.003 | 0.906 | -0.048 | 0.070 | -0.029 | 0.274 |
| **IgA** | 0.026 | 0.324 | -0.006 | 0.828 | 0.055 | 0.039 | 0.014 | 0.606 |
| **IgG** | -0.131 | <0.001 | 0.003 | 0.901 | -0.089 | <0.001 | -0.224 | <0.001 |
| **IgM** | -0.215 | <0.001 | -0.055 | 0.038 | -0.024 | 0.357 | -0.156 | <0.001 |
| **C3** | 0.016 | 0.544 | 0.188 | <0.001 | -0.045 | 0.087 | -0.047 | 0.074 |
| **C4** | 0.057 | 0.031 | 0.111 | <0.001 | -0.014 | 0.585 | 0.019 | 0.481 |

**Supplementary Table 6 Ordered logistic regression models for combination of PIV and VAS on the risk of SPP deterioration**

| **Outcome** | **Exposure** | **Model 1** | | **Model 2** | | **Model 3** | |
| --- | --- | --- | --- | --- | --- | --- | --- |
|  |  | **OR(95%CI)** | **P value** | **OR(95%CI)** | **P value** | **OR(95%CI)** | **P value** |
| PF | PIV and VAS, P for trend |  | <0.001 |  | <0.001 |  | <0.001 |
|  | Low PIV and low VAS | Ref |  | Ref |  | Ref |  |
|  | High PIV and low VAS | 2.03(1.56, 2.64) | <0.001 | 2.02(1.55, 2.64) | <0.001 | 1.82(1.38, 2.40) | <0.001 |
|  | Low PIV and high VAS | 4.38(3.29, 5.82) | <0.001 | 4.48(3.37, 5.96) | <0.001 | 4.51(3.37, 6.05) | <0.001 |
|  | High PIV and high VAS | 5.88(4.52, 7.64) | <0.001 | 5.68(4.37, 7.40) | <0.001 | 5.17(3.90, 6.85) | <0.001 |
| BP | PIV and VAS, P for trend |  | <0.001 |  | <0.001 |  | <0.001 |
|  | Low PIV and low VAS | Ref |  | Ref |  | Ref |  |
|  | High PIV and low VAS | 1.35(1.04, 1.76) | 0.024 | 1.34(1.03, 1.74) | 0.031 | 1.20(0.91, 1.58) | 0.204 |
|  | Low PIV and high VAS | 3.17(2.40, 4.20) | <0.001 | 3.17(2.40, 4.40) | <0.001 | 3.36(2.52, 4.47) | <0.001 |
|  | High PIV and high VAS | 4.68(3.62, 6.06) | <0.001 | 4.60(3.55, 5.97) | <0.001 | 4.29(3.25, 5.66) | <0.001 |
| VT | PIV and VAS, P for trend |  | <0.001 |  | <0.001 |  | <0.001 |
|  | Low PIV and low VAS | Ref |  | Ref |  | Ref |  |
|  | High PIV and low VAS | 1.31(1.01, 1.71) | 0.040 | 1.33(1.02, 1.73) | 0.033 | 1.30(0.99, 1.71) | 0.060 |
|  | Low PIV and high VAS | 3.05(2.31, 4.03) | <0.001 | 3.09(2.34, 4.09) | <0.001 | 3.14(2.36, 4.18) | <0.001 |
|  | High PIV and high VAS | 4.99(3.86, 6.46) | <0.001 | 5.00(3.85, 6.48) | <0.001 | 5.08(3.84, 6.71) | <0.001 |
| SF | PIV and VAS, P for trend |  | <0.001 |  | <0.001 |  | <0.001 |
|  | Low PIV and low VAS | Ref |  | Ref |  | Ref |  |
|  | High PIV and low VAS | 1.33(1.02, 1.73) | 0.032 | 1.32(1.02, 1.72) | 0.037 | 1.17(0.89, 1.55) | 0.261 |
|  | Low PIV and high VAS | 4.47(3.34, 5.97) | <0.001 | 4.53(3.38, 6.06) | <0.001 | 4.68(3.47, 6.31) | <0.001 |
|  | High PIV and high VAS | 6.78(5.17, 8.90) | <0.001 | 6.66(5.06, 8.75) | <0.001 | 6.20(4.64, 8.30) | <0.001 |
| MH | PIV and VAS, P for trend |  | <0.001 |  | <0.001 |  | <0.001 |
|  | Low PIV and low VAS | Ref |  | Ref |  | Ref |  |
|  | High PIV and low VAS | 0.89(0.68, 1.15) | 0.358 | 0.89(0.68, 1.15) | 0.361 | 0.84(0.64, 1.10) | 0.213 |
|  | Low PIV and high VAS | 2.36(1.79, 3.11) | <0.001 | 2.40(1.82, 3.16) | <0.001 | 2.57(1.93, 3.41) | <0.001 |
|  | High PIV and high VAS | 3.60(2.79,4.64) | <0.001 | 3.58(2.77, 4.63) | <0.001 | 3.63(2.75, 4.79) | <0.001 |
| CPRI-RA | PIV and VAS, P for trend |  | <0.001 |  | <0.001 |  | <0.001 |
|  | Low PIV and low VAS | Ref |  | Ref |  | Ref |  |
|  | High PIV and low VAS | 1.71(1.32, 2.23) | <0.001 | 1.68(1.29, 2.19) | <0.001 | 1.51(1.14, 1.99) | 0.004 |
|  | Low PIV and high VAS | 3.04(2.30, 4.01) | <0.001 | 3.05(2.31, 4.03) | <0.001 | 2.98(2.24, 3.96) | <0.001 |
|  | High PIV and high VAS | 8.45(6.47, 11.02) | <0.001 | 8.22(6.29, 10.74) | <0.001 | 7.41(5.57, 9.85) | <0.001 |
| SDS | PIV and VAS, P for trend |  | <0.001 |  | <0.001 |  | <0.001 |
|  | Low PIV and low VAS | Ref |  | Ref |  | Ref |  |
|  | High PIV and low VAS | 1.14(0.88, 1.49) | 0.319 | 1.13(0.87, 1.47) | 0.374 | 1.04(0.78, 1.37) | 0.804 |
|  | Low PIV and high VAS | 2.92(2.22, 3.86) | <0.001 | 2.95(2.23, 3.89) | <0.001 | 2.83(2.13, 3.77) | <0.001 |
|  | High PIV and high VAS | 4.43(3.44,5.71) | <0.001 | 4.30(3.33, 5.55) | <0.001 | 3.89(2.96, 5.12) | <0.001 |
| SDH | PIV and VAS, P for trend |  | <0.001 |  | <0.001 |  | <0.001 |
|  | Low PIV and low VAS | Ref |  | Ref |  | Ref |  |
|  | High PIV and low VAS | 1.49(1.15, 1.94) | 0.003 | 1.50(1.16, 1.96) | 0.002 | 1.38(1.05, 1.82) | 0.022 |
|  | Low PIV and high VAS | 2.60(1.98, 3.43) | <0.001 | 2.62(1.99, 3.45) | <0.001 | 2.27(1.72, 3.01) | <0.001 |
|  | High PIV and high VAS | 3.86(3.00, 4.97) | <0.001 | 3.92(3.04, 5.05) | <0.001 | 3.29(2.51, 4.31) | <0.001 |
| SDSSD | PIV and VAS, P for trend |  | <0.001 |  | <0.001 |  | <0.001 |
|  | Low PIV and low VAS | Ref |  | Ref |  | Ref |  |
|  | High PIV and low VAS | 1.51(1.15, 1.96) | 0.003 | 1.50(1.15, 1.96) | 0.003 | 1.40(1.06, 1.85) | 0.017 |
|  | Low PIV and high VAS | 2.55(1.93, 3.36) | <0.001 | 2.55(1.93, 3.36) | <0.001 | 2.24(1.69, 2.97) | <0.001 |
|  | High PIV and high VAS | 4.40(3.42, 5.68) | <0.001 | 4.36(3.37, 5.63) | <0.001 | 3.68(2.81, 4.84) | <0.001 |

**Supplementary Table 7 Generalized additive models for combination of PIV and VAS on the risk of SPP deterioration**

| **Outcome** | **Low PIV and**  **low VAS** | **High PIV and**  **low VAS** | | **Low PIV and**  **high VAS** | | **High PIV and**  **high VAS** | |
| --- | --- | --- | --- | --- | --- | --- | --- |
|  |  | **OR(95%CI)** | **P value** | **OR(95%CI)** | **P value** | **OR(95%CI)** | **P value** |
| PF | Ref | 2.52(1.71, 3.71) | <0.001 | 3.51(2.34, 5.25) | <0.001 | 7.79(4.83, 12.56) | <0.001 |
| BP | Ref | 1.57(0.94, 2.63) | 0.083 | 2.16(1.29, 3.64) | 0.004 | 2.20(1.32, 3.67) | 0.002 |
| VT | Ref | 1.40(1.00, 1.96) | 0.052 | 2.61(1.82, 3.74) | <0.001 | 4.54(3.11, 6.61) | <0.001 |
| SF | Ref | 0.95(0.65, 1.40) | 0.804 | 5.74(4.03, 8.18) | <0.001 | 7.03(4.98, 9.92) | <0.001 |
| RE | Ref | 1.67(1.16, 2.42) | 0.006 | 4.57(2.88, 7.28) | <0.001 | 4.90(3.16, 7.59) | <0.001 |
| MH | Ref | 0.77(0.55, 1.06) | 0.107 | 2.62(1.84, 3.73) | <0.001 | 3.28(2.31, 4.67) | <0.001 |
| CPRI-RA | Ref | 1.78(1.28, 2.47) | <0.001 | 3.40(2.44, 4.74) | <0.001 | 9.61(6.80, 13.59) | <0.001 |
| SDS | Ref | 1.52(0.82, 2.81) | 0.180 | 1.83(1.00, 3.36) | 0.051 | 5.04(2.35, 10.81) | <0.001 |
| SDH | Ref | 1.48(1.07, 2.04) | 0.017 | 2.21(1.60, 3.05) | <0.001 | 4.31(3.10, 5.99) | <0.001 |
| SDSSD | Ref | 1.56(1.13, 2.17) | 0.008 | 2.11(1.53, 2.92) | <0.001 | 4.22(3.05, 5.83) | <0.001 |

**Supplementary Table 8 Evaluation and comparison of standard Logistic regression model and generalized additive model**

| **Outcome** | **Standard Logistic Model** | | **Generalized Additive Model** | |
| --- | --- | --- | --- | --- |
|  | **AIC** | **BIC** | **AIC** | **BIC** |
| PF | 1270.45 | 1365.18 | 1273.62 | 1404.78 |
| BP | 947.22 | 1041.95 | 940.21 | 1096.50 |
| VT | 1598.25 | 1692.98 | 1579.09 | 1719.67 |
| SF | 1640.00 | 1734.73 | 1635.67 | 1766.77 |
| RE | 1296.69 | 1391.41 | 1289.50 | 1440.98 |
| MH | 1714.13 | 1808.86 | 1697.87 | 1874.19 |
| CPRI-RA | 1728.83 | 1823.55 | 1728.55 | 1881.25 |
| SDS | 642.89 | 737.62 | 639.31 | 775.24 |
| SDH | 1799.09 | 1893.82 | 1797.21 | 1932.49 |
| SDSSD | 1822.31 | 1917.04 | 1819.16 | 1987.36 |

**Supplementary Table 9 Baseline characteristics of the training and validation cohorts**

| **Characteristic** | **Training cohort**  **(n=998, 70.0%)** | **Testing cohort**  **(n=428, 30.0%)** | **P value** |
| --- | --- | --- | --- |
| PIV | 293.30 (162.46, 518.84) | 285.16 (155.81, 545.57) | 0.779 |
| VAS (cm) | 6.20 (5.50, 7.00) | 6.10 (5.50, 7.00) | 0.799 |
| Gender, n (%) |  |  | 0.561 |
| Male | 180(18.0) | 71(16.6) |  |
| Female | 818(82.0) | 357(83.4) |  |
| Age (years) | 58.00 (51.00, 68.00) | 58.00 (52.00, 68.00) | 0.935 |
| BMI (kg/m^2^) | 22.22 (20.30, 24.02) | 22.20 (20.31, 23.75) | 0.659 |
| Disease duration (years) | 9.21 (3.60, 15.54) | 9.43 (4.00, 16.00) | 0.957 |
| CCI (score) | 4.00 (3.00, 6.00) | 5.00 (3.00, 6.00) | 0.153 |
| ESR (mm/h) | 36.00 (18.00, 57.00) | 36.00 (16.00, 55.25) | 0.273 |
| Hs-CRP (mg/L) | 12.62 (3.36, 36.46) | 11.06 (2.84, 32.31) | 0.117 |
| RF (KIU/L) | 104.20 (38.62, 252.17) | 106.35 (40.95, 274.05) | 0.651 |
| CCP (U/ml) | 86.60 (16.52, 234.52) | 94.90 (12.28, 301.75) | 0.463 |
| IgA (g/L) | 2.82 (2.07, 3.75) | 2.79 (2.04, 3.72) | 0.454 |
| IgG (g/L) | 11.61 (9.44, 14.47) | 11.54 (9.41, 14.30) | 0.749 |
| IgM (g/L) | 1.24 (0.91, 1.69) | 1.28 (0.90, 1.84) | 0.237 |
| C3 (g/L) | 1.22 (1.08, 1.38) | 1.22 (1.09, 1.35) | 0.819 |
| C4 (g/L) | 0.30 (0.24, 0.37) | 0.29 (0.24, 0.37) | 0.285 |
| PF (score), n (%) |  |  | 0.467 |
| <50 | 811(81.3) | 340(79.4) |  |
| ≥50 | 187(18.7) | 88(20.6) |  |
| RP (score), n (%) |  |  | 0.126 |
| <50 | 875(87.7) | 388(90.7) |  |
| ≥50 | 123(12.3) | 40(9.3) |  |
| BP (score), n (%) |  |  | 0.512 |
| <50 | 897(89.9) | 379(88.6) |  |
| ≥50 | 101(10.1) | 49(11.4) |  |
| GH (score), n (%) |  |  | 0.371 |
| <50 | 943(94.5) | 410(95.8) |  |
| ≥50 | 55(5.5) | 18(4.2) |  |
| VT (score), n (%) |  |  | 0.328 |
| <50 | 722(72.3) | 298(69.6) |  |
| ≥50 | 276(27.7) | 130(30.4) |  |
| SF (score), n (%) |  |  | 0.747 |
| <50 | 388(38.9) | 171(40.0) |  |
| ≥50 | 610(61.1) | 257(60.0) |  |
| RE (score), n (%) |  |  | 0.141 |
| <50 | 825(82.7) | 339(79.2) |  |
| ≥50 | 173(17.3) | 89(20.8) |  |
| MH (score), n (%) |  |  | 0.367 |
| <50 | 679(68.0) | 280(65.4) |  |
| ≥50 | 319(32.0) | 148(34.6) |  |
| CPRI-RA (score), n (%) |  |  | 0.862 |
| <9.855 | 497(49.8) | 216(50.5) |  |
| ≥9.855 | 501(50.2) | 212(49.5) |  |
| SAS (score), n (%) |  |  | 0.974 |
| <50 | 266(26.7) | 113(26.4) |  |
| ≥50 | 732(73.3) | 315(73.6) |  |
| SDS (score), n (%) |  |  | 0.646 |
| <50 | 64(6.4) | 24(5.6) |  |
| ≥50 | 934(93.6) | 404(94.4) |  |
| SDH (score), n (%) |  |  | 1.000 |
| <15 | 433(43.4) | 185(43.2) |  |
| ≥15 | 565(56.6) | 243(56.8) |  |
| SDSSD (score), n (%) |  |  | 0.669 |
| <14 | 499(50.0) | 208(48.6) |  |
| ≥14 | 499(50.0) | 220(51.4) |  |
| SBS (score), n (%) |  |  | 0.445 |
| <6 | 480(48.1) | 216(50.5) |  |
| ≥6 | 518(51.9) | 212(49.5) |  |

**Supplementary Table 10 The best hyperparameters for each outcome model in 5-fold cross-validation**

| **Outcome** | **eta** | **max_depth** | **gamma** | **subsample** | **colsample_bytree** | **min_child_weight** | **nrounds** | **CV_AUC** |
| --- | --- | --- | --- | --- | --- | --- | --- | --- |
| PF | 0.1 | 7 | 0 | 0.7 | 0.7 | 1 | 119 | 0.7282 |
| RP | 0.1 | 3 | 0.2 | 0.8 | 0.8 | 3 | 34 | 0.761 |
| BP | 0.1 | 5 | 0.2 | 0.8 | 0.7 | 1 | 107 | 0.6763 |
| GH | 0.1 | 3 | 0 | 0.7 | 0.7 | 3 | 54 | 0.7992 |
| VT | 0.01 | 3 | 0 | 0.8 | 0.7 | 3 | 40 | 0.7271 |
| SF | 0.01 | 3 | 0.2 | 0.7 | 0.8 | 3 | 25 | 0.7927 |
| RE | 0.1 | 3 | 0.1 | 0.7 | 0.7 | 3 | 25 | 0.7128 |
| MH | 0.1 | 7 | 0.2 | 0.9 | 0.8 | 3 | 17 | 0.7532 |
| CPRI-RA | 0.01 | 5 | 0 | 0.8 | 0.7 | 1 | 10 | 0.7574 |
| SAS | 0.01 | 7 | 0 | 0.8 | 0.8 | 3 | 23 | 0.6804 |
| SDS | 0.1 | 7 | 0.1 | 0.8 | 0.7 | 1 | 11 | 0.6936 |
| SDH | 0.01 | 7 | 0 | 0.9 | 0.7 | 1 | 83 | 0.7156 |
| SDSSD | 0.1 | 3 | 0.1 | 0.7 | 0.8 | 3 | 20 | 0.7326 |
| SBS | 0.01 | 3 | 0.1 | 0.7 | 0.7 | 1 | 34 | 0.6061 |

## Supplementary Figures


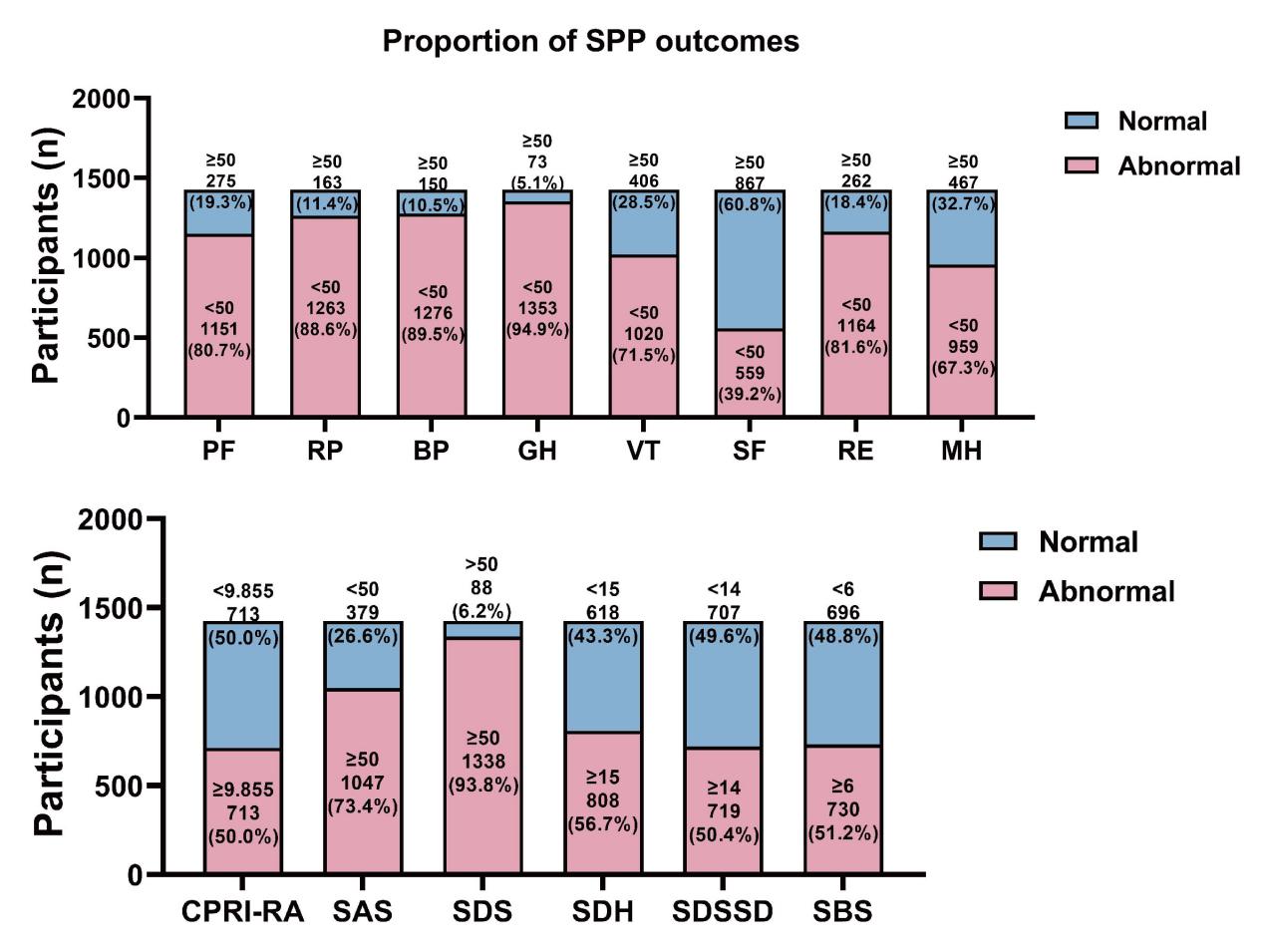


**Supplementary Figure 1 Distribution of SPP outcomes in the study population**


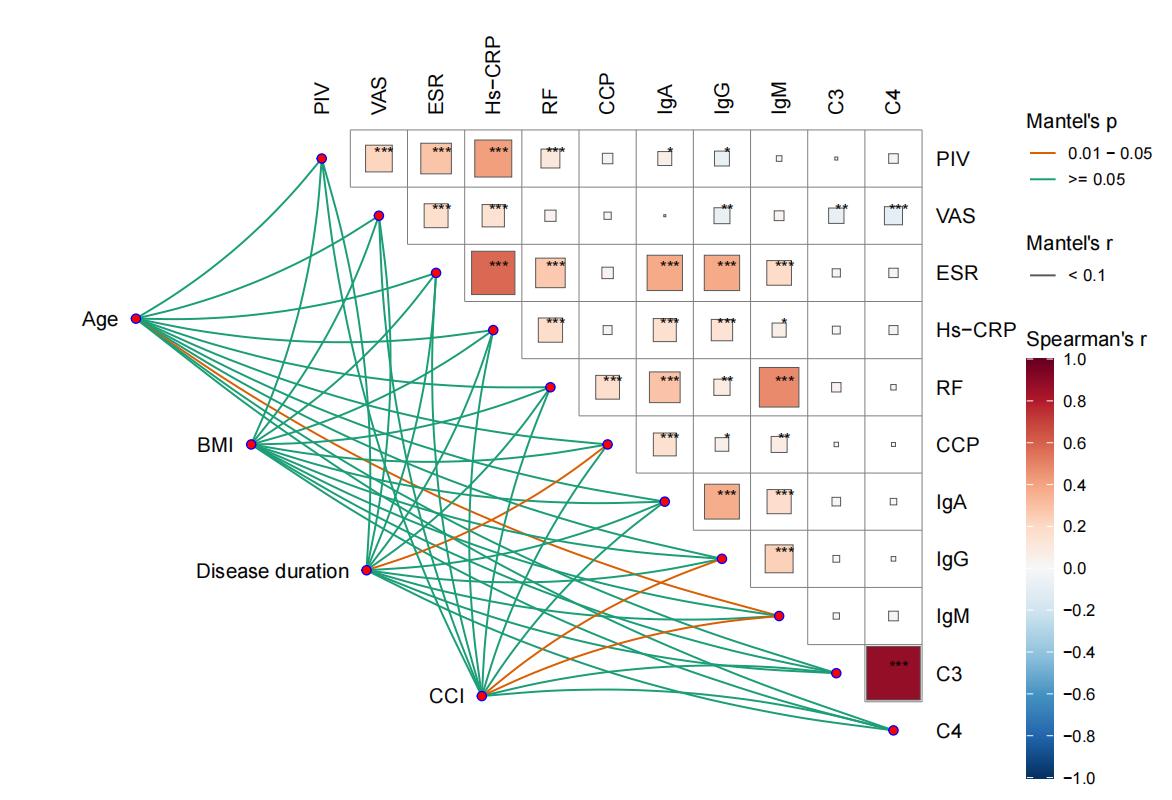


**Supplementary Figure 2 Spearman's correlation test among selected features**


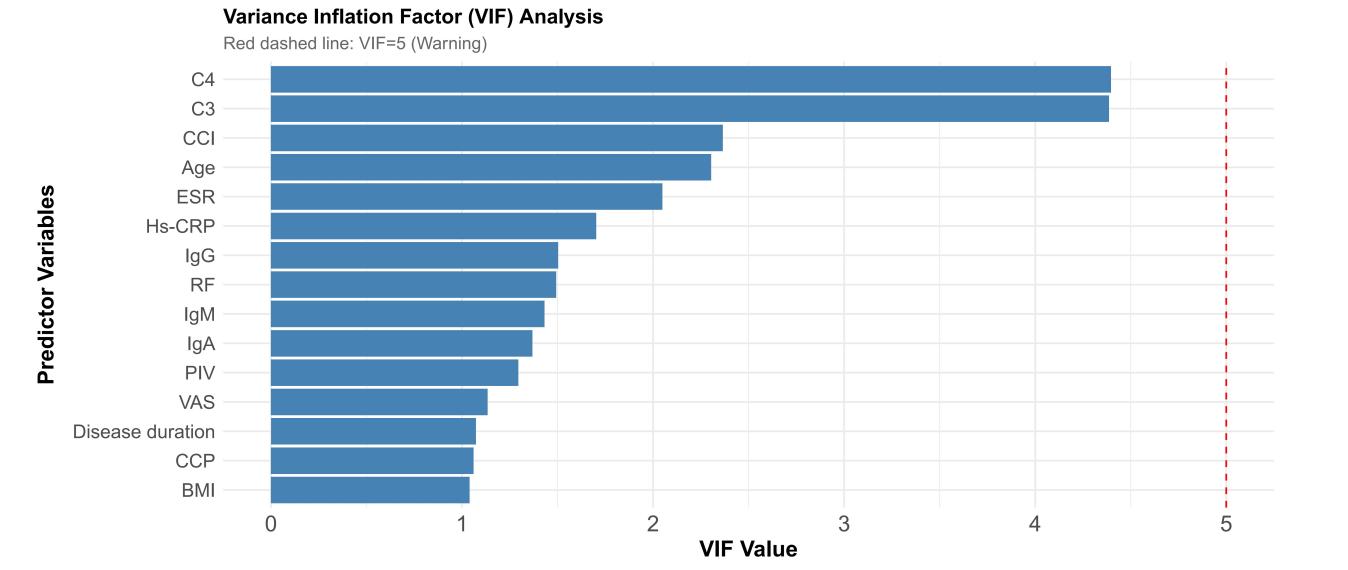


**Supplementary Figure 3 Variance inflation factor test among selected features**

**
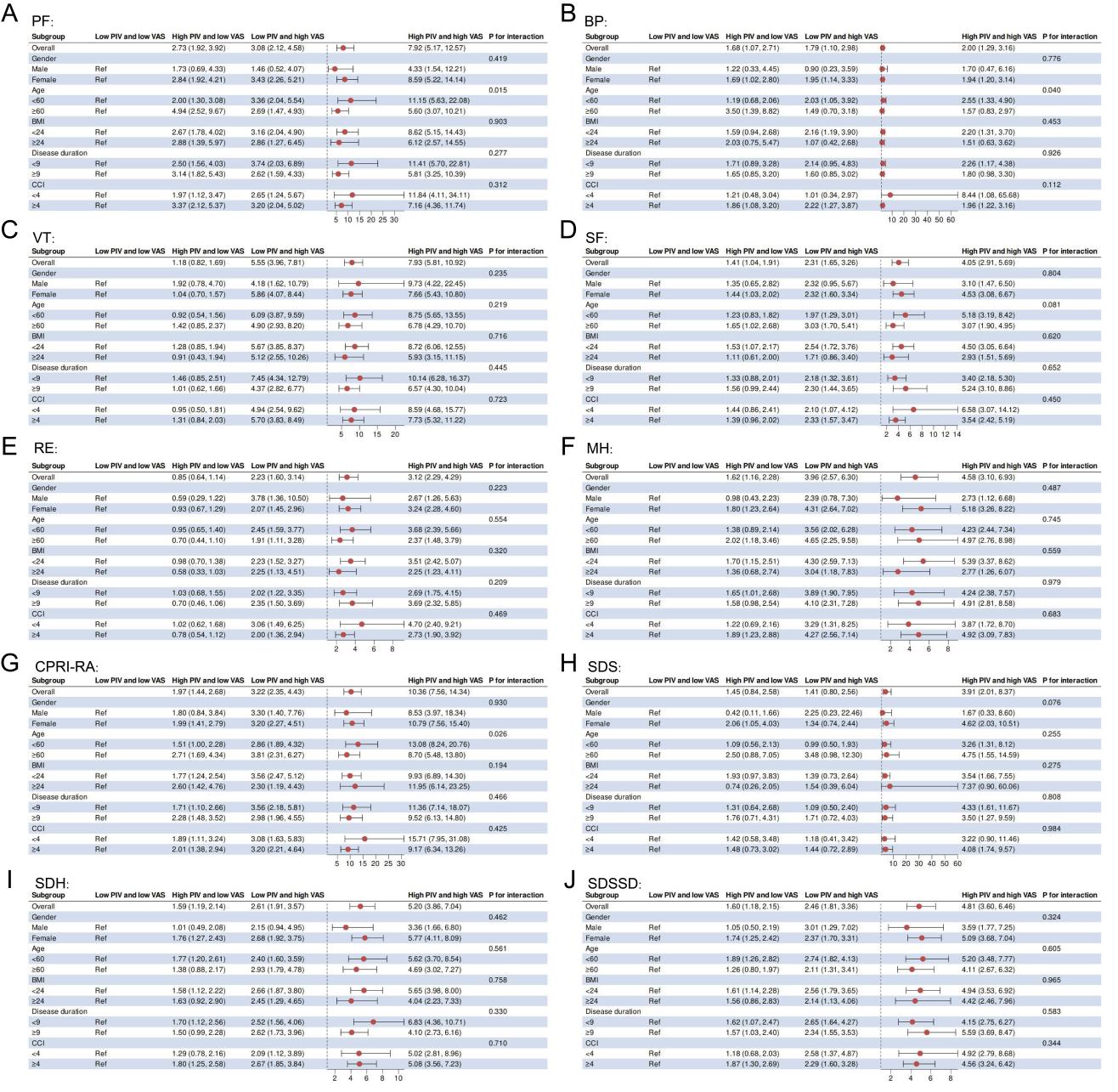
**

**Supplementary Figure 4 Subgroup analysis of the combined effect of PIV and VAS on the risk of SPP deterioration**
